# A common antigenic motif recognized by naturally occurring human V_H_5–51/V_L_4–1 anti-tau antibodies with distinct functionalities

**DOI:** 10.1186/s40478-018-0543-z

**Published:** 2018-05-31

**Authors:** Adrian Apetri, Rosa Crespo, Jarek Juraszek, Gabriel Pascual, Roosmarijn Janson, Xueyong Zhu, Heng Zhang, Elissa Keogh, Trevin Holland, Jay Wadia, Hanneke Verveen, Berdien Siregar, Michael Mrosek, Renske Taggenbrock, Jeroenvan Ameijde, Hanna Inganäs, Margot van Winsen, Martin H. Koldijk, David Zuijdgeest, Marianne Borgers, Koen Dockx, Esther J. M. Stoop, Wenli Yu, Els C. Brinkman-van der Linden, Kimberley Ummenthum, Kristof van Kolen, Marc Mercken, Stefan Steinbacher, Donata de Marco, Jeroen J. Hoozemans, Ian A. Wilson, Wouter Koudstaal, Jaap Goudsmit

**Affiliations:** 1 Janssen Prevention Center, Janssen Pharmaceutical Companies of Johnson & Johnson, Archimedesweg 6, 2333 CN Leiden, the Netherlands; 2 Janssen Prevention Center, Janssen Pharmaceutical Companies of Johnson & Johnson, 3210 Merryfield Row, San Diego, CA 92121 USA; 3 Janssen Neuroscience Discovery, Janssen Pharmaceutical Companies of Johnson & Johnson, Turnhoutseweg 30, 2340 Beerse, Belgium; 4 Molecular and Cellular Pharmacology, Discovery Sciences, Janssen Pharmaceutical Companies of Johnson & Johnson, Turnhoutseweg 30, 2340 Beerse, Belgium; 5 Department of Integrative Structural and Computational Biology, The Scripps Research Institute, La Jolla, CA 92037 USA; 6 Proteros Biostructures GmbH, Bunsenstraße 7a, 82152 Planegg, Germany; 7 Department of Pathology, Amsterdam Neuroscience, VU University Medical Center, De Boelelaan 1117, 1081 HV Amsterdam, the Netherlands; 8 Skaggs Institute for Chemical Biology, The Scripps Research Institute, La Jolla, CA 92037 USA; 9 Department of Epidemiology, Harvard T.H. Chan School of Public Health, 677 Huntington Avenue, Boston, MA 02115 USA; 10 Department of Neurology, Amsterdam Neuroscience, Academic Medical Center, Meidreefberg 9, 1105 AZ Amsterdam, the Netherlands; 11 Present address: Janssen R&D US, 3210 Merryfield Row, San Diego, CA 92121 USA; 12 Janssen Vaccines and Prevention, Janssen Pharmaceutical Companies of Johnson and Johnson, Archimedesweg 6, Leiden, CN 2333 the Netherlands

**Keywords:** Alzheimer’s disease, Tau protein, Monoclonal antibody, Antigenic motif

## Abstract

Misfolding and aggregation of tau protein are closely associated with the onset and progression of Alzheimer’s Disease (AD). By interrogating IgG^+^ memory B cells from asymptomatic donors with tau peptides, we have identified two somatically mutated V_H_5–51/V_L_4–1 antibodies. One of these, CBTAU-27.1, binds to the aggregation motif in the R3 repeat domain and blocks the aggregation of tau into paired helical filaments (PHFs) by sequestering monomeric tau*.* The other, CBTAU-28.1, binds to the N-terminal insert region and inhibits the spreading of tau seeds and mediates the uptake of tau aggregates into microglia by binding PHFs. Crystal structures revealed that the combination of V_H_5–51 and V_L_4–1 recognizes a common Pro-X_n_-Lys motif driven by germline-encoded hotspot interactions while the specificity and thereby functionality of the antibodies are defined by the CDR3 regions. Affinity improvement led to improvement in functionality, identifying their epitopes as new targets for therapy and prevention of AD.

**Electronic supplementary material:**

The online version of this article (10.1186/s40478-018-0543-z) contains supplementary material, which is available to authorized users.

## Introduction

Intracellular neurofibrillary tangles (NFTs) consisting of aggregated tau protein are a hallmark of Alzheimer’s disease (AD) and other neurogenerative disorders, collectively referred to as tauopathies [31]. Tau is a microtubule-associated protein expressed predominantly in neuronal axons and promotes the assembly and stability of microtubules [9, 46]. It is expressed in the adult human brain as six isoforms with zero, one or two N-terminal acidic inserts (0N, 1N, or2 N) and either three or four microtubule-binding repeats (3R or 4R) [18]. The tau protein contains many potential phosphorylation sites and the regulated phosphorylation and dephosphorylation of several of these has been shown to affect its interaction with tubulin and cytoskeleton function [4, 38]. Hyperphosphorylation of tau is thought to lead to microtubule dissociation and assembly of the normally disordered, highly soluble protein into β sheet-rich aggregated fibrils called paired helical filaments (PHFs) that make up NFTs [8, 30, 34]. While the molecular mechanism of tau aggregation remains elusive, it is believed that its initial nucleation step is energetically unfavorable, whereas the subsequent fibril growth follows an energetically downhill landscape [2, 11, 23, 39]. Accumulating evidence indicates that these fibrils can transmit from cell to cell and spread tau pathology to distant brain regions by seeding the recruitment of soluble tau into *de novo* aggregates [16, 21, 22, 26, 47].

**Fig. 1:**
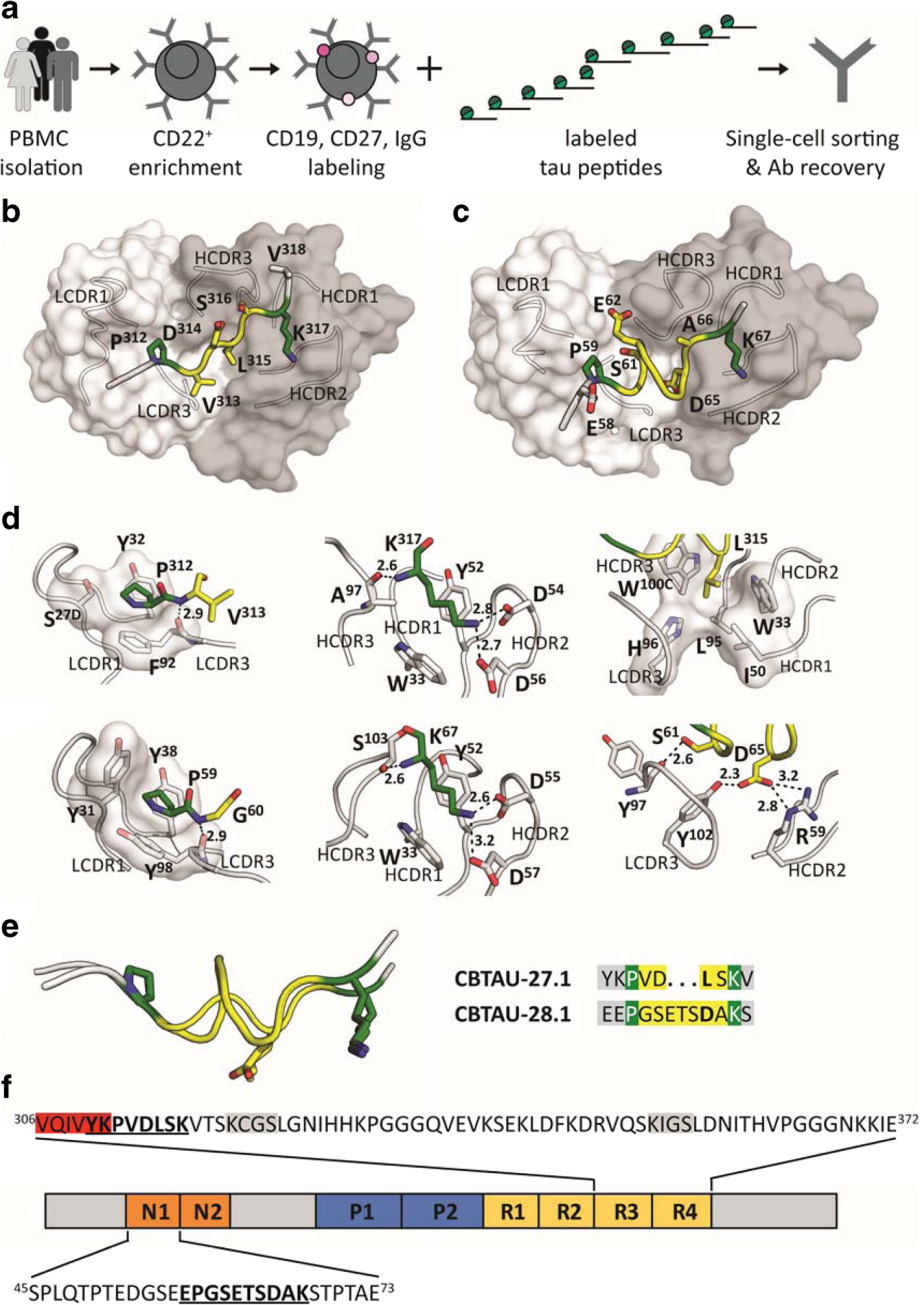
Recovery and structural characterization of naturally occurring monoclonal antibodies to unphosphorylated tau epitopes from asymptomatic individuals. **a** BSelex method used to recover tau-specific memory B cells. PBMCs were prepared from asymptomatic blood bank donors, and mature CD22^+^ B cells were positively selected with magnetic beads. Viable cells were stained with IgG-FITC, CD19-PerCPCy5.5, and CD27-PECy7, and with a pool of 10 overlapping unphosphorylated tau peptides spanning the longest tau isoform (relative position of each peptide along 2N4R tau indicated). All peptides were present in the pool with an APC label as well as with a PE label and CD19^+^, CD27^+^, IgG^+^, APC^+^, PE^+^ cells were single-cell sorted on a Beckman Coulter MoFlo XDP. Antibody heavy and light variable chain sequences were recovered from single cells, cloned and expressed as full-length IgGs. **b** and **c** Co-crystal structures of Fab CBTAU-27.1 (**b**) and Fab CBTAU-28.1 (**c**) with tau peptides A8119 and A7731, respectively. Antibodies have been plotted as molecular surface with light chain in white and heavy chain in grey. Tau peptides are shown as cartoon with interacting amino acids plotted as sticks. Proline and lysine residues are plotted in green, amino acids in between these residues are colored in yellow and the termini in grey. Only the interacting antibody loops are outlined. **d** Key interactions with tau of CBTAU-27.1 (upper row) and CBTAU-28.1 (lower row). Key interacting residues are plotted as sticks, polar interactions are indicated with dotted lines, and the corresponding distances are indicated in Å. In the first panel, interactions with Pro^312^ and Pro^59^ are compared where the proline binding pockets are visualized on a molecular surface. In the second panel, interactions with Lys^317^ and Lys^67^ are compared. In panel 3, interactions around Leu^315^ and Asp^65^ in the central region of the epitopes are shown. **e** Structural basis for recognition of the Pro – X_n_ – Lys epitope motif. Epitopes of CBTAU-27.1 and CBTAU-28.1 are superimposed by aligning on the proline and lysine residues. The peptides present both residues in the same spatial orientation. In the central region (yellow), hydrophobic Leu^315^ in CBTAU-27.1 is replaced by hydrophilic and charged Asp^65^ in CBTAU-28.1. **f** Schematic representation of tau isoform 2N4R showing the epitopes of CBTAU-27.1 and CBTAU  -28.1 (bold and underlined) and the surrounding sequences. Highlighted in grey and red are the microtubule binding motifs and the hexapeptide ^306^VQIVYK^311^ which forms the N-terminal end of the core of PHFs, respectively. N1 and N2 indicate acidic inserts, P1 and P2 indicate proline-rich domains, and R1-R4 indicate microtubule-binding repeat domains

Several monoclonal antibodies that inhibit the spreading of tau fibrils have been described and are being developed for antibody-based therapies [6, 42, 48, 49]. We have recently described the isolation of a panel of antibodies with such functional activity by interrogating the peripheral IgG^+^ memory B cells of healthy human blood donors for reactivity to phosphorylated tau peptides [37]. To expand the arsenal of potential targets and include epitopes present in physiological tau, we used the BSelex technology in combination with a pool of unphosphorylated tau peptides as baits (Fig. 1a, and Additional file 1: Table S1 for peptide sequences).

## Materials and methods

### Human PBMC isolation

Whole blood from healthy male and female donors was obtained from the San Diego Blood Bank (ages 18–65 years) after informed consent was obtained from the donors. PBMCs were isolated on Ficoll-Paque Plus (GE Healthcare) and cryopreserved at 50 million cells per ml in 90% FBS and 10% DMSO.

### Single cell sorting and recovery of heavy and light variable light antibody genes from tau-specific memory B cells

Tau-peptide specific B cells were sorted and antibody chains were recovered as previously described [37]. Briefly, a panel of 10 biotinylated peptides spanning the longest tau isoform, tau441 (Additional file 1: Table S1) were mixed with streptavidin-APC or streptavidin-PE (Thermo Fisher) at a 35:1 molar ratio and free peptide removed using BioSpin 30 column (Biorad). PBMCs corresponding to 3 donors were thawed and B cells were enriched by positive selection with CD22^+^ magnetic beads (Miltenyi Biotec). B cells were subsequently labeled with extracellular markers IgG-FITC, CD19-PerCPCy5.5 and CD27-PECy7 (all from BD Biosciences) and incubated with labeled peptide baits. Doublets and dead cells were excluded and the CD19^+^, IgG^+^, CD27hi, antigen double-positive cells were collected by single cell sorting into PCR plates containing RT-PCR reaction buffer and RNaseOUT (Thermo Fisher). Heavy and light chain (HC/LC) antibody variable regions were recovered using a two-step PCR approach from single sorted memory B cells using a pool of leader specific (Step I) and framework specific primers (Step II PCR). Heavy and light chain PCR fragments (380–400 kb) were linked via an overlap extension PCR and subsequently cloned into a dual-CMV–based human IgG1 mammalian expression vector (generated in house). Finally, all recovered antibodies were expressed and tested by ELISA against the tau peptides used in the sorting experiments to identify tau specific antibodies.

### Peptide synthesis

All peptides were synthesized at Pepscan BV (Lelystad, The Netherlands) or New England Peptide, Inc. using routine Fmoc-based solid phase strategies on automated synthesizers. After acidic cleavage and deprotection, peptides were purified by reversed phase HPLC, lyophilized and stored as powders at − 80 °C. Purity and identity were confirmed by LC-MS.

### Recombinant IgG expression and purification

Human IgG1 antibodies were constructed by cloning the heavy (V_H_) and light (V_L_) chain variable regions into a single expression vector containing the wildtype IgG constant regions. Plasmids encoding the sequences corresponding to human anti-tau mAbs were transiently transfected in human embryonic kidney 293-derived Expi293F™ cells (Thermo Fisher) and 7 days post transfection, the expressed antibodies were purified from the culture medium by MabSelect SuRe (GE Healthcare) Protein A affinity chromatography. IgGs were eluted from the column with 100 mM sodium citrate buffer, pH 3.5 which was immediately buffer exchanged into PBS, pH 7.4 using a self-packed Sephadex G-25 column (GE Healthcare). Mouse chimeric versions of CBTAU-27.1 and CBTAU-28.1 were generated cloning the variable light and heavy chain into an expression vector in which the human Fc was replaced with murine Fc. Fab fragments were obtained by papain digestion of antibodies (Thermo Fisher Scientific), followed by removal of the Fc using MabSelect SuRe resin (GE Healthcare). Each antibody was quality controlled by SDS-PAGE and size exclusion chromatography coupled with multi angle light scattering (SEC-MALS) and was further confirmed for reactivity to cognate tau peptide by Octet biolayer interferometry.

### Recombinant tau (rtau) expression and purification

The gene encoding the human tau-441 isoform (2N4R) extended with an N-terminal His-tag and a C-terminal C-tag and containing the C291A and C322A mutations was cloned into a kanamycin resistant bacterial expression vector and transformed into BL21 (DE3) cells. A 3 ml 2-YT Broth (Invitrogen) preculture containing 25 mg/l kanamycin was inoculated from a single bacterial colony for 6 h after which it was diluted to 300 ml for overnight growth in a 1-l shaker flask and subsequently diluted to 5 -l in a 10-l wave bag. Protein expression was induced when the culture reached an OD_600nm_ 1.0 by addition of 2 mM IPTG. Three hours after induction, the bacterial pellets were harvested by centrifugation, and lysed with Bugbuster protein extraction reagent (Merck) supplemented with a protease inhibitor cocktail (cOmplete™ ULTRA tablets EDTA free, Roche). Purification was performed by affinity chromatography using self-packed Ni-Sepharose (GE Healthcare) and C-Tag resin (Thermo Fisher Scientific) columns.

### Reactivity of anti-tau human mAbs to tau peptides by ELISA

Reactivity of recovered mAbs were tested against biotinylated tau peptides as previously described [37]. Briefly, tau peptides were captured on streptavidin-coated plates (Thermo Fisher Scientific) at 1 μg/ml in TBS and incubated for 2 h. Goat anti-human Fab (2 μg/ml, Jackson ImmunoResearch) to measure total IgG was used, and bovine actin (1 μg/ml TBS, Sigma) and irrelevant peptide were used to confirm specificity of the purified mAbs. ELISA plates were blocked and purified IgGs were diluted to 5 μg/ml in TBS/0.25% BSA and titrated (5-fold serial dilutions) against peptides. Plates were subsequently washed and goat anti-human IgG F(ab’)2 (Jackson ImmunoResearch) was used at 1:2000 dilution as secondary. Following incubation, plates were washed four times in TBS-T and developed with Sure Blue Reserve TMB Microwell Peroxidase Substrate (KPL) for approximately 2 min. The reaction was stopped by the addition of TMB Stop Solution and the absorbance at 450 nm was measured using an ELISA plate reader.

### Western blot analysis

Immunopurified PHF and sarkosyl-insoluble fraction of AD brain lysates were provided by Steven Paul at Weill Cornell Medical College and prepared as described previously [20]. Approximately 0.3 μg protein was resolved on SDS-PAGE (4–12% Bis-Tris Novex NuPAGE gel; Invitrogen) and subsequently transferred onto a nitrocellulose membrane. The membrane was blocked overnight in 1X TBS-T with 5% BSA (blocking buffer). CBTAU-27.1 and CBTAU-28.1 were used at 25 μg/ml in 2.5% BSA in TBS-T and incubated for 2 h at room temperature. The membranes were then washed three times for 5 min each in TBS-T. Peroxidase AffiniPure goat anti-human IgG (Fc-γ fragment specific; Jackson ImmunoResearch) was used as secondary antibody at a 1:4000 dilution in 2.5% BSA in TBS-T and incubated for 1 h at room temperature. The membrane was washed three times for 5 min and developed using the Supersignal West Pico kit (Pierce). Images were obtained on the ImageQuant LAS-4000 (GE Healthcare).

### Qualitative association and dissociation measurements by Octet biolayer interferometry

The relative binding of the antibodies to tau peptides was assessed by biolayer interferometry (Octet Red 384) measurements (ForteBio) [10]. Biotinylated tau peptides were immobilized on Streptavidin (SA) Dip and Read biosensors for kinetics (ForteBio). Real-time binding curves were measured by applying the sensor in a solution containing 100 nM antibody. To induce dissociation, the biosensor containing the antibody-tau peptide complex was immersed in assay buffer without antibody. The immobilization of peptides to sensors, the association and the dissociation steps, were followed in different ionic strength buffers containing 10% FortéBio kinetics buffer as assay buffer. The relative association and dissociation kinetic curves were compared to qualitatively assess the efficiency of antibody binding to peptides encompassing different tau epitopes.

### Affinity measurements by Isothermal Titration Calorimetry (ITC)

The affinities of antibodies for their corresponding tau peptides were determined in solution on a MicroCal Auto-iTC200 system (Malvern). Peptides at concentrations of ~ 35 μM (CBTAU-27.1), ~ 10 μM (dmCBTAU-27.1), ~ 30 μM (CBTAU-28.1) and ~ 33 μM (dmCBTAU-28.1) were titrated in 20 steps of 2 μl per step, except for dmCBTAU-27.1 where 40 steps of 1 μl were employed, in identical buffers containing 200 μM CBTAU-27.1, 130 μM dmCBTAU-27.1, 205 μM CBTAU-28.1 and 205 μM dmCBTAU-28.1, respectively. The thermodynamic parameters and the equilibrium dissociation constants, Kd, were determined upon fitting the ITC data to a model assuming a single set of binding sites corresponding to an antibody:tau peptide = 1:2 binding model.

### Expression, crystallization, data collection, structure determination, and refinement of CBTAU-27.1 Fab and CBTAU-28.1 Fab

To express Fab fragments for crystallization, the gene sequences coding for the hinge, C_H_2, and C_H_3 of human antibodies CBTAU-27.1 (IgG1*κ*) and CBTAU-28.1 (IgG1*κ*) were removed from the corresponding IgG expression vector and a His_6_ tag was added to the C-terminus of each C_H_1. Both Fabs were produced by transient transfection of FreeStyle 293-F cells and purified using a Ni-NTA column followed by a size exclusion chromatography using a Superdex 75 or Superdex 200 column (GE Healthcare). The purified CBTAU-27.1 and CBTAU-28.1 Fabs were concentrated to ~ 10 mg/ml and ~ 8 mg/ml, respectively, in 20 mM Tris, pH 8.0 and 150 mM NaCl for crystallization. Crystallization experiments were set up using the sitting drop vapor diffusion method. Initial crystallization conditions for CBTAU-27.1 Fab and its complex with peptide 2833–1 (HVPGGGSVQIVYKPVDLSKV), and CBTAU-28.1 in complex with peptide W1805 (TEDGSEEPGSETSDAKSTPT-amide) were obtained from robotic crystallization trials using the robotic CrystalMation system (Rigaku) at The Scripps Research Institute. For co-crystallization, CBTAU-27.1 and CBTAU-28.1 Fabs were mixed with peptides 2833–1 and W1805, respectively, in a molar ratio of 1:10 before screening. Crystals of *apo*-form CBTAU-27.1 Fab were obtained at 20 °C from a reservoir solution containing 0.1 M Hepes, pH 7.5, 20% PEG8000, while crystals of CBTAU-27.1 Fab with 2833–1 were grown at 20 °C from 0.1 M Hepes, pH 7.0, 0.1 M KCl, 15% PEG5000 MME. Crystals of CBTAU-28.1 with W1805 were obtained at 20 °C from 0.085 M Tris-HCl, pH 8.5, 0.17 M sodium acetate, 25.5% PEG4000. Before data collection, the crystals of CBTAU-27.1 Fab and CBTAU-28.1 Fab were soaked in the reservoir solution supplemented with 25% (v/v) and 15% (v/v) glycerol, respectively, for a few seconds and then flash-frozen in liquid nitrogen. X-ray diffraction data of CBTAU-27.1 Fab *apo*-form were collected to 1.9 Å resolution at beamline 23ID-D at the Advanced Photon Source (APS). X-ray diffraction data of CBTAU-27.1 Fab with 2833–1 and CBTAU-28.1 with W1805 were collected to 2.0 and 2.1 Å resolution, respectively, at beamline 12–2 at the Stanford Synchrotron Radiation Lightsource (SSRL). HKL2000 (HKL Research, Inc.) was used to integrate and scale the diffraction data (Additional file 1: Table S2). The structures were determined by molecular replacement with Phaser [35] using search models of a human antibody 2-23b3 Fab (PDB ID 3QOS) for CBTAU-27.1 Fab and a human antibody 1-69b3 (PDB ID 3QOT) for CBTAU-28.1 Fab. The models were iteratively rebuilt using Coot [14] and refined in Phenix [1]. Refinement parameters included rigid body refinement and restrained refinement including TLS refinement. Electron density for both peptides 2833–1 and W1805 were clear and the peptides were built in the later stages of the refinement. Final refinement statistics are summarized in Additional file 1: Table S2.

### Crystallization, data collection, and structure determination of dmCBTAU-27.1 Fab and dmCBTAU-28.1 Fab

For crystallization, the dmCBTAU-27.1 and dmCBTAU-28.1 Fab fragments (in 20 mM HEPES buffer, pH 7.5, 7.55 mM NaCl) were incubated with 4 mM of the respective peptide on ice overnight and concentrated to a final concentration of about 50 mg/ml. The Fab:peptide complexes (dmCBTAU-27.1 Fab with tau peptide A8119 and dmCBTAU-28.1 Fab with tau peptide A7731) were subjected to crystallization screening by sitting drop vapor diffusion testing 2300 conditions, by mixing 0.1 μl protein solution and 0.1 μl reservoir, and also varying the protein concentration. The dmCBTAU-27.1 Fab with peptide A8119 was crystallized from 0.10 M sodium citrate buffer, pH 5.0, 20.00% (w/v) PEG 8 K at a concentration of 17 mg/ml. The dmCBTAU-28.1 Fab with peptide A7731 was crystallized from 22% (w/v) PEG5K MME, 0.10 M HEPES buffer, pH 6.75, 0.20 M KCl at a concentration of 50 mg/ml. For cryo-protection, crystals were briefly immersed in a solution consisting of 75% reservoir and 25% glycerol. X-ray diffraction data were collected at temperature of 100 K at the Swiss Light Source. Data were integrated, scaled and merged using XDS [25]. The structure was solved with MOLREP [43] and refined with REFMAC5 [44]. Manual model completion was carried out using Coot [14]. The quality of the final model was verified PROCHECK [29] and the validation tools available through Coot [14]. The dmCBTAU-27.1 diffraction data were indexed in space group C2 and the dmCBTAU-28.1 data in space group P2_1_2_1_2_1_. Data were processed using the programs XDS and XSCALE (see Additional file 1: Table S3). The phase information necessary to determine and analyze the structure was obtained by molecular replacement using previously solved structure of dmCBTAU-27.1 and dmCBTAU-28.1 Fabs were used search models. Subsequent model building and refinement was performed according to standard protocols with the software packages CCP4 and COOT. For calculation of R-free, 6.2% of the measured reflections were excluded from refinement (see Additional file 1: Table S3). TLS refinement (using REFMAC5, CCP4) resulted in lower R-factors and higher quality of the electron density map for dmCBTAU-27.1-A8119 using five TLS groups. Waters were added using the “Find waters” algorithm of COOT into Fo-Fc maps contoured at 3.0 sigma followed by refinement with REFMAC5 and checking all waters with the validation tool of COOT. The occupancy of side chains, which were in negative peaks in the Fo-Fc map (contoured at − 3.0 σ), were set to zero and subsequently to 0.5 if a positive peak occurred after the next refinement cycle. The Ramachandran plot of the final model shows 88.7% (dmCBTAU-27.1) and 88.1% (dmCBTAU-28.1) of all residues in the most favored region, 10.8% (dmCBTAU-27.1) and 11.6% (dmCBTAU-28.1) in the additionally allowed region, and 0.3% (dmCBTAU-27.1) and 0.0% (dmCBTAU-28.1) in the generously allowed region. Residue Ala51(L) is found in the disallowed region of the Ramachandran plot (Additional file 1: Table S3) for both Fabs, as is frequently found in other Fabs [3] . This was either confirmed from the electron density map or could not be modelled in another more favorable conformation. Statistics of the final structure and the refinement process are listed in Additional file 1: Table S3.

### Affinity maturation by phage display

The coding sequence for scFv directed against CBTAU-28.1 epitope was cloned into an inducible prokaryotic expression vector containing the phage M13 pIII gene. Random mutations were introduced in the scFv by error prone PCR (Genemorph II EZClone Domain Mutagenesis kit) after which the DNA was transformed into TG1bacteria. The transformants were grown to mid-log phase and infected with CT helper phages that have a genome lacking the infectivity domains N1 and N2 of protein pIII, rendering phage particles which are only infective if they display the scFv linked to the full-length pIII [28]. Phage libraries were screened using magnetic beads coated with rtau in immunotubes. To deselect nonspecific binders, the tubes were coated with a tau peptide lacking the CBTAU-28.1 epitope. To ensure maturation against the correct epitope, selection was continued using beads coated with the cognate A6940 peptide. Eluted phages were used to infect XL1-blue F′ *E. coli* XL1-blue F′ which were cultured and infected with CT-helper (or VCSM13) phages to rescue phages used for subsequent selection rounds. After three rounds of panning, individual phage clones were isolated and screened in phage ELISA for binding to rtau and cognate CBTAU-28.1 peptide A6940.

### In vitro tau aggregation assay

Stock solutions of 500 μM thioflavin T (ThT) (Sigma-Aldrich, St Louis, MO, USA) and 55 μM heparin (Mw = 17–19 kDa; Sigma-Aldrich, St Louis, MO, USA) were prepared by dissolving the dry powders in reaction buffer (0.5 mM TCEP in PBS, pH 6.7), and filtered through a sterile 0.22 μm pore size PES membrane filter (Corning, NY, USA) or a sterile 0.22 μm pore size PVDF membrane filter (Merck Millipore, Tullagreen, Cork, IRL), respectively. The concentration of the ThT solution was determined by absorption measurements at 411 nm using an extinction coefficient of 22,000 M^− 1^ cm^− 1^. The huTau441 concentration was determined by absorption measurements at 280 nm using an extinction coefficient of 0.31 ml mg^− 1^ cm^− 1^. For spontaneous conversions, mixtures of 15 μM huTau441 in 200 μl reaction buffer containing 8 μM heparin and 50 μM ThT were dispensed in 96-well plates (Thermo Scientific, Vantaa, Finland) that were subsequently sealed with plate sealers (R&D Systems, Minneapolis, MN). For seeding experiments, preformed seeds were added to the wells before sealing the plate. To assess the effect of IgG or Fab on the conversion, huTau441 and IgG or Fab were mixed and incubated for 20 min in reaction buffer before the addition of heparin and ThT. Kinetic measurements were monitored at 37 °C in a Biotek Synergy Neo2 Multi-Mode Microplate Reader (Biotek, VT, USA) by measuring ThT fluorescence at 485 nm (20 nm bandwidth) upon excitation at 440 nm (20 nm bandwidth) upon continuous shaking (425 cpm, 3 mm).

### Atomic force microscopy

For each sample, 20 μl of rtau solution was deposited onto freshly cleaved mica surface. After 3 min incubation, the surface was washed with double-distilled water and dried with air. Samples were imaged using the Scanasyst-air protocol using a MultiMode 8-HR and Scanasyst-air silicon cantilevers (Bruker Corporation, Santa Barbara, USA). Height images of 1024 × 1024 pixels in size and surface areas of 10 × 10 μm were acquired under ambient environmental conditions with peak force frequency of 2 KHz.

### Assessment of CBTAU-27.1 binding to rtau and PHFs by SEC-MALS

15 μM monomer or aggregated rtau were incubated with dmCBTAU-27.1 in a rtau:IgG1 = 1:0.6 ratio for 15 min and samples were subsequently centrifuged for 15 min at 20,000 g. Same procedure was also applied for controls containing only monomer rtau, aggregated rtau or IgG. All samples were analyzed by SEC-MALS upon fractionation on a **TSKgel G3000SWxl** (Tosoh Bioscience) gel filtration column equilibrated with 150 mM sodium phosphate, 50 mM sodium chloride at pH 7.0. at a flow rate of 1 ml/min. For molar mass determination, in-line UV (Agilent 1260 Infinity MWD, Agilent Technologies), refractive index (Optilab T-rEX, Wyatt Technology) and 8-angle static light scattering (Dawn HELEOS, Wyatt Technology) detectors were used.

### Immunohistochemistry

Brain samples were obtained from The Netherlands Brain Bank (NBB), Netherlands Institute for Neuroscience, Amsterdam. All donors had given written informed consent for brain autopsy and the use of material and clinical information for research purposes. Neuropathological diagnosis was assessed using histochemical stains (haematoxylin and eosin, Bodian and/or Gallyas silver stains [41] and immunohistochemistry for Amyloid beta, p-tau (AT8), α-synuclein, TDP-43 and P62, on formalin-fixed and paraffin-embedded tissue from different parts of the brain, including the frontal cortex (F2), temporal pole cortex, parietal cortex (superior and inferior lobule), occipital pole cortex, amygdala and the hippocampus, essentially CA1 and entorhinal area of the parahippocampal gyrus. Staging of pathology was assessed according to Braak and Braak for tau pathology and Thal for Amyloid beta pathology [5, 40]. Formalin-fixed and paraffin embedded tissue sections (5 μm thick) were mounted on Superfrost Plus tissue slides (Menzel-Gläser, Germany) and dried overnight at 37 °C. Sections were deparaffinised and subsequently immersed in 0.3% H_2_O_2_ in phosphate-buffered saline (PBS) for 30 min to quench endogenous peroxidase activity. Sections were either treated in sodium citrate buffer (10 mM sodium citrate, pH 6.0) heated by autoclave (20 min at 130 °C) for antigen retrieval or processed without heat pretreatment. Between the subsequent incubation steps, sections were washed extensively with PBS. Primary antibodies were diluted in antibody diluent (Immunologic) and incubated overnight at 4 °C. Secondary EnVison™ HRP goat anti-rabbit/mouse antibody (EV-GαM^HRP^, DAKO) was incubated for 30 min at room temperature (RT). 3,3-Diaminobenzidine (DAB; DAKO) was used as chromogen. Sections were counterstained with haematoxylin to visualize the nuclei of the cells, dehydrated and mounted using Quick-D mounting medium (BDH Laboratories Supplies, Poole, England). For the Gallyas silver staining, 30 μm thick sections were rinsed in distilled water and incubated in 5% periodic acid for 30 min at RT, followed by an incubation in silver iodide solution (4% sodium hydroxide, 10% potassium iodide and 0.35% silver nitrate in distilled water) for 30 min at RT. Subsequently, sections were washed in 0.5% acetic acid and developed with developer working solution (10 volumes 5% sodium carbonate solution, 3 volumes solution 0.2% ammonium nitrate, 0.2% silver nitrate and 1% Tungstosilicic acid solution, and 7 volumes 0.2% ammonium nitrate, 0.2% silver nitrate, 1% Tungstosilicic acid and 0.3% formaldehyde solution. After color development, sections were rinsed in 0.5% acetic acid, after which sections were incubated in 5% sodium thiosulphate and rinsed in distilled water. Stained sections were mounted on coated glass slides (Menzel-Gläser) and dried for at least 2 h at 37 °C. Subsequently sections were fixed in ethanol 70% for 10 min, counterstained with hematoxylin, dehydrated and mounted with Quick D mounting medium.

### FRET based cellular immunodepletion assay

Cryopreserved brain tissue was acquired from the Newcastle Brain Tissue Resource biobank. Frozen brain tissue samples from 17 AD patients were homogenized in homogenization buffer (10 mM Tris (Gibco), 150 mM NaCl (Gibco) containing protease inhibitors (cOmplete™ ULTRA tablets EDTA free, Roche) to obtain a 10% (w/v) pooled brain homogenate. Individual antibody dilutions were prepared in PBS pH 7.4 (Sigma), mixed with brain extract in a 1:1 ratio in a 96 well PCR plate (Thermo Scientific), and incubated until the beads were washed. Protein-G DynaBeads (Life Technologies) were added in a 96-well PCR plate (Thermo Scientific) and washed twice with PBS, 0.01% Tween20 (Sigma) by pulling down the beads with a magnet (Life Technologies). Wash buffer was removed completely and 10 μl of PBS, 0.1% Tween20 were added to the beads together with 90 μl of the 1:1 antibody-brain extract mixture. Samples were incubated over night at 4 °C, rotating at 5 rpm. The following day, the immunodepleted fractions were separated from the beads by pulling down the beads with the magnet, transferred to a new 96-well PCR plate and stored at − 80 °C until tested. Each condition was tested in duplicate. Immunodepleted fractions were incubated for 10 min with Lipofectamine 2000 (Invitrogen) in Opti-MEM (Gibco) in a 96-well cell culture plate (Greiner Bio-one) before 5.5 × 10^3^ HEK biosensor cells (provided by M. Diamond, Washington University School of Medicine) were added to each well. After a 2-day incubation at 37 °C, cells were washed twice with PBS, detached using Trypsin/EDTA (Gibco) and transferred to a polypropylene round bottom plate (Costar) containing FACS buffer (Hank’s Balanced Salt Solution (Sigma), 1 mM EDTA (Invitrogen), 1% FBS (Biowest)). Cells were then analyzed for FRET positivity by flow cytometry using a FACS Canto II (BD Bioscience). Each plate contained a brain extract only condition (to assess baseline FRET response) and an antibody isotype control. Results are reported as normalized values, relative to condition without antibody.

### Microglia assay

Aggregated recombinant 2N4R tau (rtau) was generated in the absence of ThT under the conditions described for the in vitro tau aggregation assay and covalently labelled with pHrodo® Green STP Ester (Invitrogen) following manufacturer’s instructions. Briefly, rtau aggregates were spun down by centrifugation at 20800 rcf for 30 min and then resuspended in 0.1 M sodium bicarbonate buffer, pH 8.5, at a final concentration of 2 mg/ml. Efficiency of aggregation was assessed by detecting presence of tau in the supernatant using SEC-MALS. Prior to labeling, tau aggregates were briefly sonicated. Ten moles of dye were added per mole of protein and the mixture was incubated for 45 min at room temperature, protected from light. Unconjugated dye was removed using a PD10 column (GE Healthcare) equilibrated with 0.1 M sodium bicarbonate buffer pH 8.5 and eluting the protein with the same buffer. Eluted fractions were evaluated for their protein content by BCA assay (Thermo Fisher Scientific) following the manufacturer’s instructions. Protein containing fractions were pooled, aliquoted and stored at − 20 °C. BV-2 cells were cultured in DMEM supplemented with 10% FBS, 100 U/ml penicillin, 100 μg/ml streptomycin and 2 mM L-Glutamine. Cultures were maintained in humidified atmosphere with 5% CO2 at 37 °C. In order to generate immunocomplexes, 250 nM aggregated rtau, covalently labelled with pHrodo Green dye, was incubated with a serial dilution (12.5–150 nM) of a chimeric version (mouse Fc region) of CBTAU-28.1 (parental and high affinity mutant) or CBTAU-27.1 (parental and high affinity mutant) in serum-free medium. Tau immunocomplexes were also generated with 300 nM Fab fragments of both CBTAU-28.1 and CBTAU-27.1, in the parental and high affinity mutant format. In each experiment, a mouse IgG1 isotype control was included together with cells incubated with only aggregated rtau. Immunocomplexes were incubated over night at 4 °C and the day after applied to BV2 cells for 2 h at 37 °C with 5% CO_2_. During the incubation, antibody-independent tau uptake was prevented by blocking the Heparan Sulfate Proteoglycan Receptor with 200 μg/ml Heparin. After incubation, cells were harvested with 0.25% trypsin-EDTA for 20 min thus simultaneously removing tau bound to the extracellular membrane, centrifuged at 400 rcf to remove medium, washed twice with PBS, and resuspended in flow cytometry buffer (PBS 1× plus 0.5% BSA and 2 mM EDTA). Cells were analyzed with a Canto II flow cytometer (BD) gating for live single cell population, as identified by forward and side scatter profiles. Results are reported as geometric mean fluorescent intensities. Each experiment was performed twice. For the microscopy experiments, cells were seeded in 96-well μClear® plate (Greiner Bio-one). After incubation with the immunocomplexes, nuclei were stained with Hoechst (Sigma) and the acidic cellular compartment with LysoTracker Red dye (Thermo Fisher). Live-cell imaging was performed using the Opera Phenix™ High Content Screening System (PerkinElmer) with temperature set to 37 °C and in presence of 5% CO_2_. For high quality images, a 63× water immersion objective was used and 0.5 μm planes (20 per Z-stack) were acquired per imaged field.

## Results

### Identification of naturally occurring anti-tau antibodies in healthy donors

In total, 2.6 × 10^6^ memory B cells from nine healthy blood donors aged 18–65 years were interrogated against a pool of 10 overlapping peptides spanning the length of tau441 (Additional file 1: Table S1). Ninety-two tau-reactive B cells were sorted and 30 heavy and light variable chain sequences were recovered and full-length IgGs were cloned and expressed. Two unique tau binding antibodies, CBTAU-27.1 and CBTAU-28.1, which are both derived from the V_H_5–51 and V_L_4–1 germline families, were identified. Both antibodies carry high numbers of somatic mutations with 38 and 28 nucleotide substitutions for the heavy and light chains of CBTAU-27.1, and 19 and 16 for the heavy and light chains of CBTAU-28.1, respectively. Since memory B cell selections were performed using a peptide pool, an ELISA-based binding assay with the 10 individual tau peptides was performed and established that CBTAU-27.1 and CBTAU-28.1 bound to peptide A6897 (residues 299–369) and peptide A6940 (residues 42–103), respectively. Further mapping narrowed the epitope regions to residues 299–318 for CBTAU-27.1 and 52–71 for CBTAU-28.1 (Additional file 1: Figure S1). The specificity of these antibodies for tau was confirmed by Western blot (Additional file 1: Figure S2).

### Recognition of a structurally identical, germline encoded hotspot motif

Crystal structures of the Fab fragments of CBTAU-27.1 and CBTAU-28.1 in complex with tau peptides spanning residues 299–318 and 52–71 were determined at 2.0 and 2.1 Å resolution, respectively (Fig. 1b and c, Additional file 1: Table S2). The structures reveal that an intriguing similarity exists in the way they bind despite recognition of very distinct regions on the tau protein (Fig. 1d). Both light chains harbor a pocket made of aromatic tyrosine or phenylalanine side chains that form a binding site for a proline residue in the N-terminal region of the different peptides. This interaction is further stabilized by a peptide backbone hydrogen bond to LCDR3 Phe^92^ (CBTAU-27.1) and LCDR3 Tyr^98^ (CBTAU-28.1). Similarly, the two heavy chains interact with a lysine in the peptide C-terminal region that involves identical hydrogen bonding networks with two HCDR2 aspartates and the backbone of HCDR3 Ala^97^ (CBTAU-27.1) and Ser^103^ (CBTAU-28.1), respectively. Both lysines are flanked by HCDR1 Trp^33^ and HCDR2 Tyr^52^ that align and stabilize the aliphatic part of the Lys side chains. However, the central parts of the tau epitopes differ significantly (see Fig. 1d, column 3). The four-residue central region adopts an extended structure in CBTAU-27.1 and inserts Leu^315^ into a pocket formed by LCDR3, HCDR3, HCDR1 and HCDR2. In the same spatial location, the seven-residue central region of the CBTAU-28.1 epitope spans the same distance between the conserved proline and lysine residues by adopting a more compact helical structure (Fig. 1e). CBTAU-28.1 Asp^65^ makes a salt bridge with Arg^59^ (HCDR2) and a hydrogen bond with Tyr^102^ (LCDR3). These two antibodies thus recognize a Pro – X_n_ – Lys motif in different tau peptides, where n is from 4 up to at least 7 amino acids. The proline and lysine binding pockets are germline-encoded and specificity towards one or other epitope arises from the CDR3 loops, which interact with the X region (Fig. 1d).

The CBTAU-27.1 epitope encompasses residues ^310^YKPVDLSK^317^ in the R3 domain (Fig. 1f). The R3 domain is part of the core of PHFs [12, 15], where a hexapeptide ^306^VQIVYK^311^ is crucial for PHF assembly [12, 15, 32, 45]. Since the CBTAU-27.1 epitope overlaps this hexapeptide, in particular the key Lys^311^ [32], we hypothesize that CBTAU-27.1 binding to tau could block the nucleation interface and thus prevent aggregation. The CBTAU-28.1 epitope encompasses residues ^58^EPGSETSDAK^67^ in the first N-terminal insert (Fig. 1f). Since the N- and C-terminal tau regions that surround the repeat domains have been shown to be disordered and project away from the PHF core to form a flexible fuzzy coat [15], binding of CBTAU-28.1 is unlikely to interfere with PHF formation, but may—like previously described antibodies [6, 42, 48, 49]—hamper the spreading of aggregates after they are formed. However, the affinities of both antibodies, at least to their cognate tau peptides, are in the high nanomolar range (Additional file 1: Figure S3A and B), which may limit their functional activity. Therefore, we set out to generate affinity-improved mutants of CBTAU-27.1 and CBTAU-28.1 by employing a combination of rational design and random mutagenesis approaches.

### Affinity-improved antibodies retain specificity

**Fig. 2:**
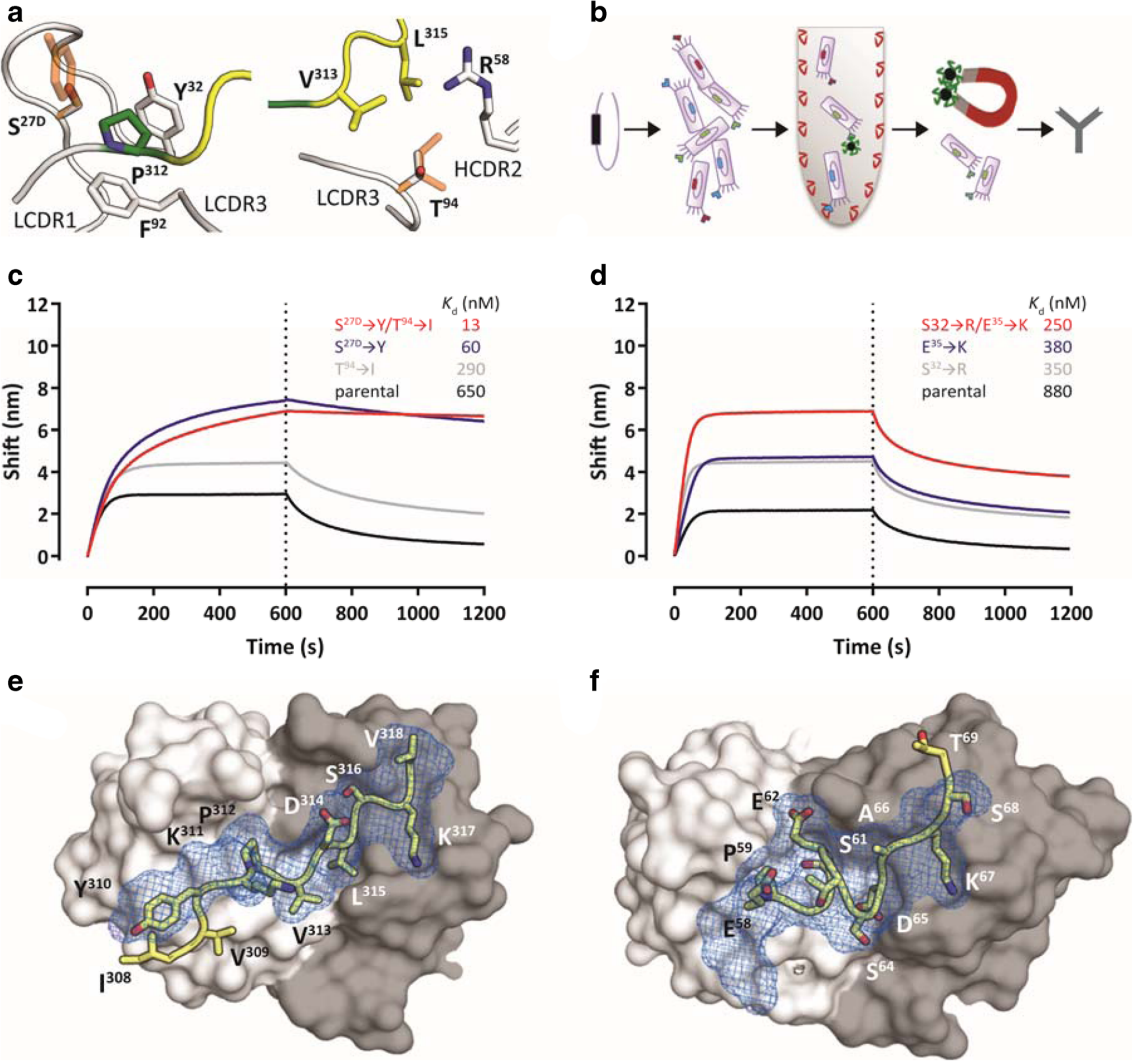
Generation of affinity-improved mutants of CBTAU-27.1 and CBTAU-28.1 **a** Structure-based design of mutants around Pro^312^ (left panel) and Val^313^ (right panel). The tau epitope is illustrated as in Fig. 1b. Antibody loops and the key residues interacting with Pro^312^ and Thr^94^ are plotted in white. Proposed mutations are shown as orange sticks on top of the corresponding wild-type side chains. Ser^27D^ is mutated to tyrosine (left panel) to enlarge the hydrophobic pocket of Pro^312^, and Thr^94^ is mutated to isoleucine (right panel) to fill the empty cavity surrounding Val^313^ and Leu^315^. By introducing both mutations, additional hydrophobic contacts between tau and the antibody loops could be formed, potentially resulting in a lower desolvation penalty and increased affinity. **b** Schematic representation of the CBTAU-28.1 affinity maturation process by random mutagenesis. Mutations were introduced randomly by error prone PCR in the coding sequence for the single-chain variable fragment (scFv) directed against the CBTAU-28.1 epitope. M13 phage libraries displaying the scFv were screened against rtau and peptide A6940. Affinity-matured variants were identified by phage ELISA and converted into an IgG1 format to assess binding in solution. **c** and **d** Association and dissociation profiles for parental and affinity improved CBTAU-27.1 (**c**) and CBTAU-28.1 (**d**) variants to their corresponding cognate peptides as determined by Octet biolayer interferometry. Affinities as determined by ITC (*K*_d_) are shown on the individual graphs. (**e** and **f**) Co-crystal structures of the Fabs of dmCBTAU-27.1 (**e**) and dmCBTAU-28.1 (**f**) with tau peptides A8119 and A7731, respectively. Antibodies are illustrated as molecular surfaces (colored as in panel A), together with tau epitopes as sticks with yellow carbons. The corresponding parental co-crystal structures have been aligned using their variable regions, and their tau epitopes are shown as blue mesh on top of the mutant epitopes.

For CBTAU-27.1, we used a rational structure-based approach through analysis of the co-crystal structure (Fig. 2a). LCDR3 Thr^94^ was identified as one location where additional hydrophobic interactions could be formed without affecting the structure of the tau peptide. Isoleucine introduced at this position better filled the gap between Val^313^, Leu^315^ and the aliphatic portion of V_H_ Arg^58^. In LCDR1, Ser^27D^ was mutated to tyrosine to remove the unfavorable contact between the serine hydroxyl and the proline pyrrolidine sidechain and create additional hydrophobic interactions. These two mutations improved the affinity by more than 50-fold to the low nanomolar range (Fig. 2c and Additional file 1: Figure S3). For CBTAU-28.1, analysis of the structure did not reveal potential affinity-improving mutations and, therefore, a random mutagenesis strategy was employed (Fig. 2b). This approach led to the identification of Ser^32^ ➔Arg and Glu^35^ ➔ Lys mutations in the light chain that combined led to an ~ 4-fold improvement in affinity compared to the parental antibody (Fig. 2d and Additional file 1: Figure S3). Co-crystal structures of the Fab fragments of the CBTAU-27.1 double mutant Ser^27D^ ➔ Tyr / Thr^94^ ➔ Ile (from here on referred to as dmCBTAU-27.1) and the CBTAU-28.1 double mutant Ser^32^ ➔ Arg / Glu^35^ ➔ Lys (from here on referred to as dmCBTAU-28.1) in complex with peptides A8119 and A7731, respectively, were determined at 3.0 and 2.85 Å resolution (Additional file 1: Table S3). Alignment of the structures of the double mutants in complex with their tau epitopes to the corresponding parental antibody co-crystal structures showed that both dmCBTAU-27.1 and dmCBTAU-28.1 retained the binding mode of the parental antibody (Fig. 2e and f), with RMSD values for the peptide Cα atoms of 0.44 Å and 0.24 Å, respectively. The similarity between the double mutants and their parental antibodies regarding the nature of their interactions with tau was confirmed by biolayer interferometry using buffers of different ionic strengths (Additional file 1: Figure S4). Furthermore, binding of the different antibodies to sets of tau peptides was assessed to confirm conservation of the specificity (Additional file 1: Figure S4).

**Fig. 3:**
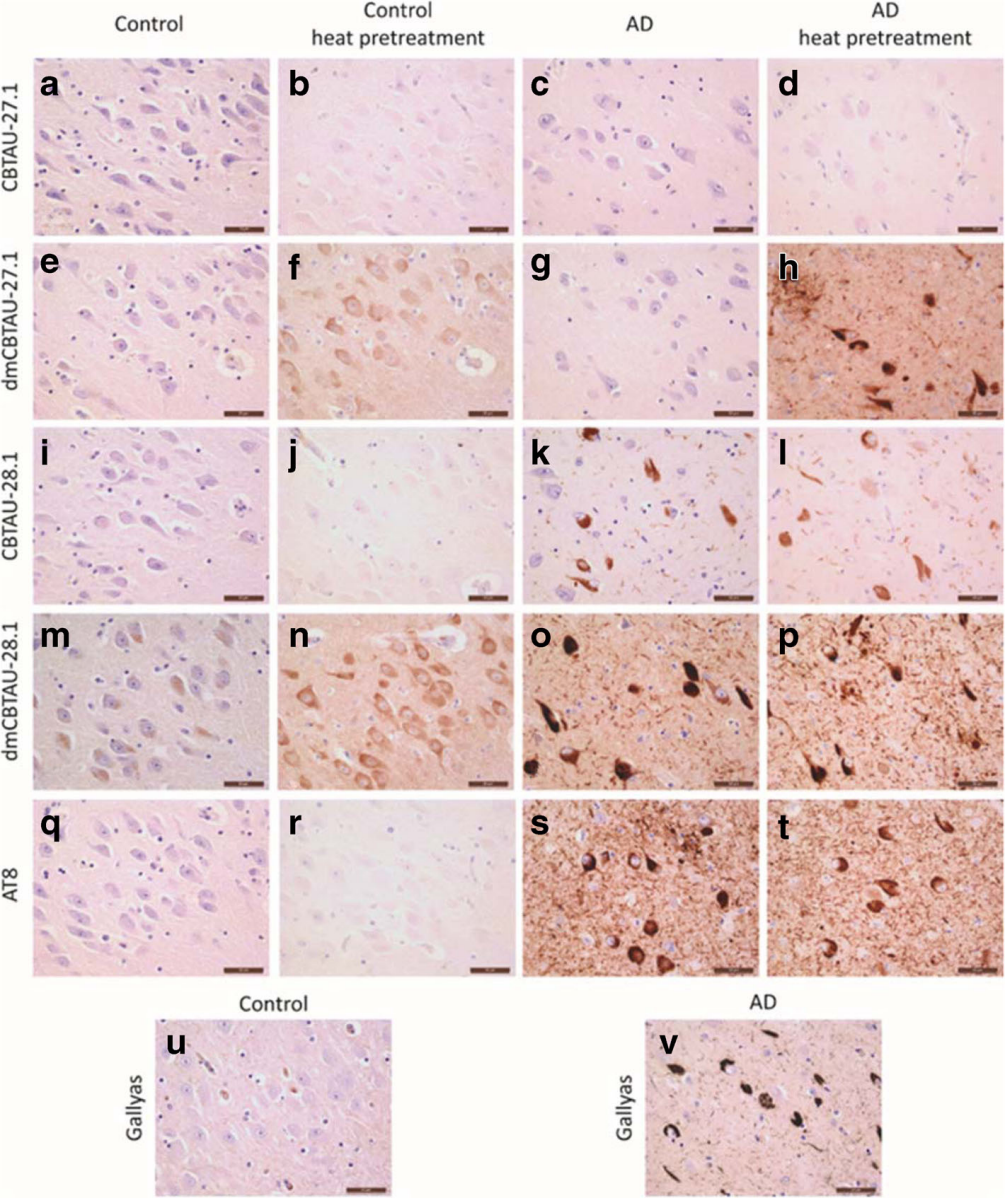
Detection of immunoreactivity in human brain tissue by CBTAU-27.1 and CBTAU-28.1 and affinity-improved variants. Immunohistochemistry was performed on 5 μm thick formalin-fixed paraffin embedded sections of the hippocampal region using a 0.1 μg/ml antibody concentration. Immunodetection using CBTAU-27.1 (**a-d**), dmCBTAU-27.1 (**e-h**), CBTAU-28.1 (**i-l**), dmCBTAU-28.1 (**m-p**) and PHF-tau-specific mouse antibody AT8 (**q-t**) in control and AD brain tissue without or with heat pretreatment using sodium citrate buffer. Gallyas staining for detection of NFTs and neuropil threads is shown from the same control and AD case of corresponding areas for comparison (**u**, **v**). Immunoreactivity was visualized using DAB (brown) and nuclei were counterstained with haematoxylin (blue). Representative areas of the CA1/subiculum of the hippocampus are shown. Scale bars represent 50 μm

The tau specificity of the antibodies was further assessed by immunohistochemical staining on post-mortem control and AD brain tissue (Fig. 3). CBTAU-27.1 did not show immunoreactivity in either control or AD cases, whereas dmCBTAU-27.1 showed immunoreactivity in the cytosol of neurons of the control cases and clear recognition of aggregated tau in neuropil threads and NFTs in AD brains, but only after heat pretreatment which is a routine ‘antigen retrieval’ procedure to recover reactivity in formalin-fixed paraffin-embedded tissue sections. No immunoreactivity of CBTAU-28.1 was detected in the control cases, whereas dmCBTAU-28.1 showed diffuse immunoreactivity of neurons after heat pretreatment. In AD brains, both CBTAU-28.1 and dmCBTAU-28.1 recognized neuropil threads and NFTs regardless of the sample treatment. CBTAU-28.1 thus recognizes PHFs without heat pretreatment whereas CBTAU-27.1, even in its high affinity variant, requires heat pretreatment to recognize pathologic tau. This is in line with the epitope of CBTAU-27.1 being buried within the PHFs and becoming exposed upon heat pretreatment. The diffuse neuronal immunoreactivity of dmCBTAU-27.1 and dmCBTAU-28.1 observed in control brain tissue after heat pretreatment shows that these antibodies bind to physiological tau. The observed immunoreactivity under identical conditions shows a clear improvement in the detection of tau by affinity-improved antibodies relative to parental antibodies without affecting specificity (Fig. 3). Similar results were obtained with immunohistochemical staining on post-mortem brain tissue of other tauopathies like frontotemporal lobar degeneration (FTLD), Pick’s disease, progressive supranuclear palsy (PSP) and primary age-related tauopathy (PART) cases (Additional file 1: Figure S5). Both CBTAU-27.1 and CBTAU-28.1 recognize pathological tau structures in all these diseases, but detection by CBTAU-27.1 is dependent on heat pretreatment to make its epitope accessible. Furthermore, the detection of tau is improved for the affinity-improved antibodies.

### Binding domain-dependent functional activities

The observation that the epitope of CBTAU-27.1 forms part of the core of the PHFs led us to consider that CBTAU-27.1 might be able to prevent aggregation of tau by inhibiting the initial nucleation step. While the molecular mechanism of tau aggregation is not fully understood, the current paradigm is that it follows a nucleation-dependent polymerization (NDP) process [2, 11, 23, 39]. An NDP mechanism is characterized by an initial nucleation step (nuclei formation) followed by an exponential growth step (fibril elongation). Nucleation, the rate-limiting step of the aggregation process, is a stochastic phenomenon and refers to the formation of high energy nuclei. Once nuclei are formed, they rapidly recruit tau monomer (growth step), and convert into thermodynamically stable aggregates. These aggregates can undergo fragmentation generating more fibril ends that are capable of recruiting tau monomers and converting them into *de novo* fibrils. This process is in most general terms referred to as “seeding”. To assess whether CBTAU-27.1 can interfere with the aggregation of tau, we have developed a robust and highly reproducible in vitro assay that monitors the heparin-induced aggregation of full-length recombinant Tau441 (rtau) by thioflavin T (ThT) fluorescence. The aggregation behavior of tau in our assay fulfills the expected features of an NDP: sigmoidal kinetic curves with a well-defined lag phase followed by exponential growth ending in a stationary phase (Additional file 1: Figure S6). The aggregation kinetics of tau were highly reproducible and displayed the expected concentration dependence (Additional file 1: Figure S6B). Furthermore, the obtained tau aggregates displayed PHF-like morphology as assessed by atomic force microscopy (AFM) (Additional file 1: Figure S6C) and were extremely efficient in seeding *de novo* aggregation of tau (Additional file 1: Figure S6D and E).

**Fig. 4:**
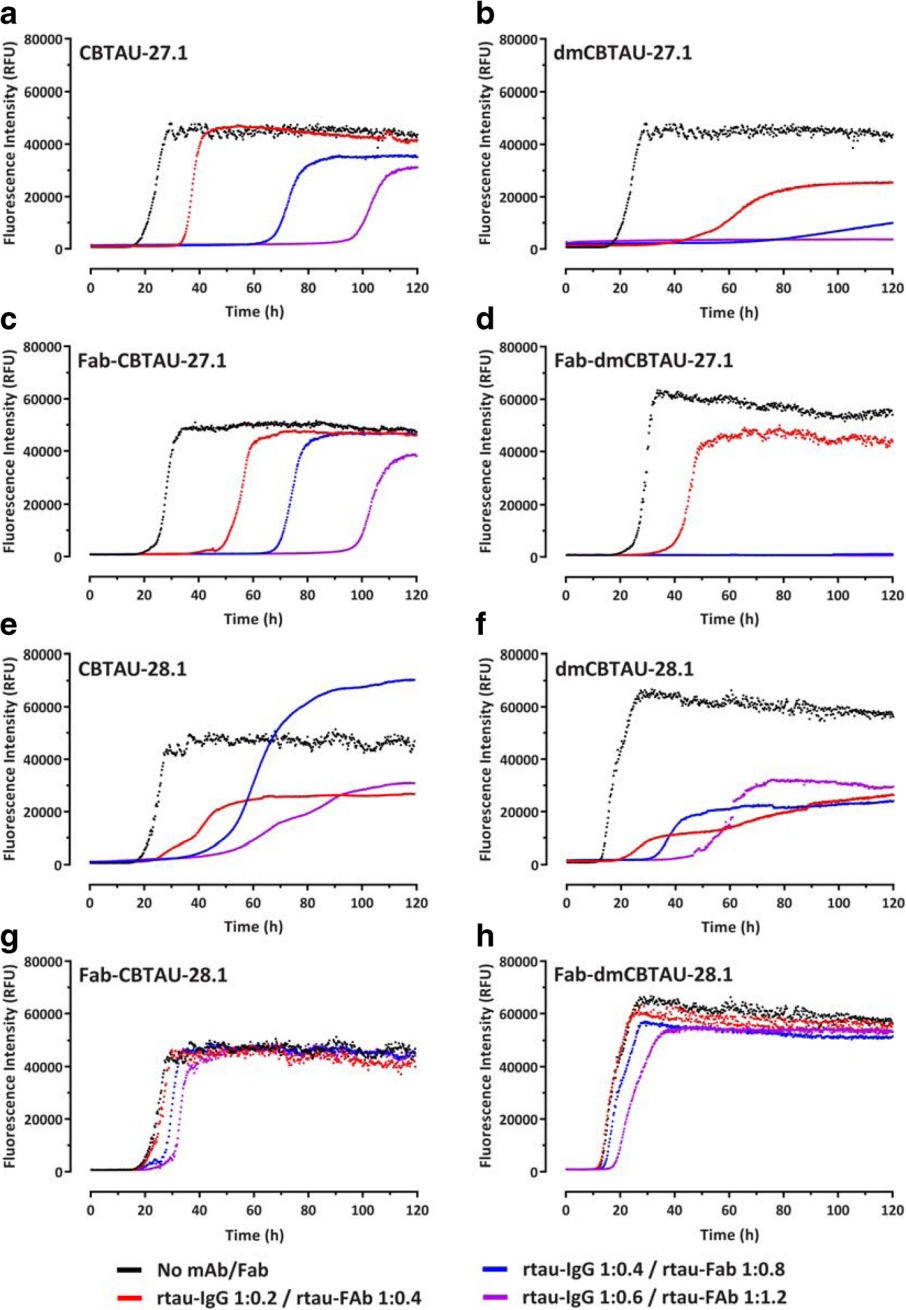
CBTAU-27.1, but not CBTAU-28.1, inhibits the aggregation of recombinant tau in vitro. Aggregation of rtau in the absence (black) or presence of CBTAU-27.1 (**a**), dmCBTAU-27.1 (**b**), Fab CBTAU-27.1 (**c**), Fab dmCBTAU-27.1 (**d**), CBTAU-28.1 (**e**), dmCBTAU-28.1 (**f**), Fab CBTAU-28.1 (**g**) or Fab dmCBTAU-28.1 (**h**), as monitored continuously for 120 h by ThT fluorescence. Three different rtau-IgG (1:0.2 – red, 1:0.4 – blue, and 1: 0.6 – purple) and rtau-Fab (1:0.4 – red, 1: 0.8 – blue, and 1:1.2 – purple) stoichiometries were tested. Each condition was tested in quadruplicate and one representative curve is shown for each condition. For complete datasets, see Additional file 1: Figures S7-S10

In agreement with our hypothesis, CBTAU-27.1 inhibited tau aggregation, as reflected by longer lag phases and lower final ThT fluorescence signal in the kinetic curves. This inhibitory effect was strongly enhanced after affinity improvement (Fig. 4a, and b, and Additional file 1: Figure S7). To shed further light on the mechanism, we assessed the ability of dmCBTAU-27.1 to alter the tau conversion when added at later times after initiating the aggregation. In all cases, our results show that stoichiometric amounts of dmCBTAU-27.1 can not only prevent tau aggregation, but also arrest it even in the exponential phase where significant amounts of seeds are already present, presumably by binding and sequestering monomeric tau (Additional file 1: Figure S11). The hypothesis that CBTAU-27.1 targets monomeric tau and does not interact with tau aggregates was further confirmed by sedimentation experiments followed by SEC-MALS size determination, which indicates that the antibody binds to two tau monomers using its two Fab arms while not being able to co-sediment with preformed tau aggregates (Additional file 1: Figure S12). In sum, these results confirm the initial hypothesis that an antibody that targets the key PHF interface of the monomeric tau can block its misfolding and aggregation.

Both double mutant and parental CBTAU-28.1 showed comparable alterations in the aggregation kinetics (Fig. 4e, and f, and Additional file 1: Figure S8). While somewhat longer lag phases and lower end-point fluorescent signals were observed in the presence of antibody, the effect did not seem to be dose dependent and the kinetics were strikingly irreproducible. Furthermore, visual inspection of reaction mixtures after 120 h incubation revealed that CBTAU-28.1 induced formation of large polymeric structures (Additional file 1: Figure S13), suggesting it can crosslink tau aggregates. This notion is supported by the fact that the Fabs of both parental and dmCBTAU-28.1 did not affect tau aggregation (Fig. 4g, and h, and Additional file 1: Figure S10). In contrast, CBTAU-27.1 and dmCBTAU-27.1 Fabs showed similar inhibitory effects as their corresponding antibodies (Fig. 4c, and d, and Additional file 1: Figure S9), emphasizing the different mechanisms by which CBTAU-27.1 and CBTAU-28.1 interfere with tau aggregation.

**Fig. 5:**
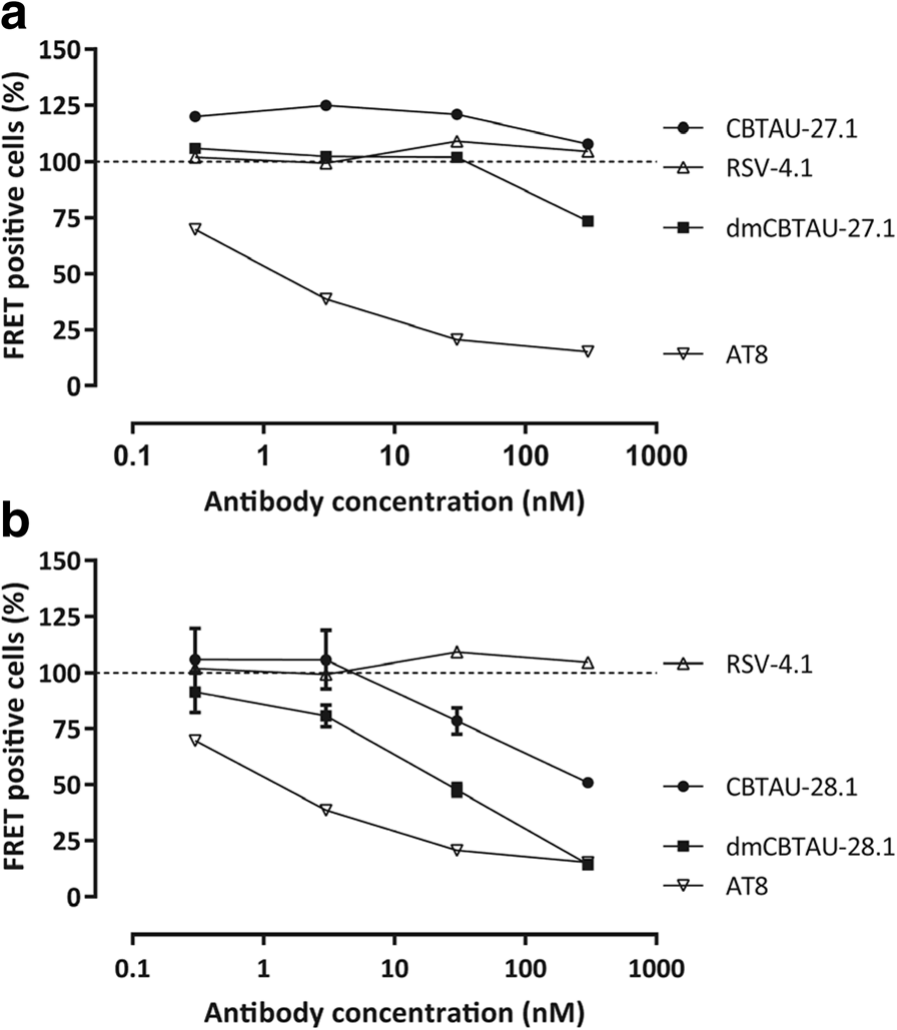
CBTAU-28.1, but not CBTAU-27.1, is capable of immunodepleting seeds from AD brains. Residual seeding activity of human AD brain homogenates following immunodepletion with different concentrations of CBTAU-27.1 and dmCBTAU-27.1 (**a**) or CBTAU-28.1 and dmCBTAU-28.1 (**b**) as measured by FRET signal in biosensor cells expressing the microtubule repeat domains of tau (aa 243–375) fused either to yellow or cyan fluorescent protein. Uptake of exogenous tau aggregates into the cells results in aggregation of the tau fusion proteins, which is detected by FRET. As positive and negative controls, a human IgG1 chimeric version of murine anti-PHF antibody AT8 and anti-RSV-G antibody RSV-4.1 were taken along, respectively. For the controls, the same data are shown in plots A and B for visualization purposes. Error bars indicate the SD of two independent experiments

We next assessed the ability of the antibodies to bind tau aggregates and thus potentially block the propagation and spreading of tau pathology. We therefore incubated human AD brain homogenate containing PHFs with the antibodies and depleted the antibody-antigen complexes. The residual seeding capacity was assessed using a cell-based biosensor assay [24, 48]. In line with its inability to bind PHFs, CBTAU-27.1 did not reduce the seeding activity of the AD brain homogenate, while dmCBTAU-27.1 showed only minor reduction and only at the highest concentration tested (Fig. 5a). In contrast, CBTAU-28.1, like mouse anti-PHF antibody AT8, depletes seeding activity from AD brain homogenate and this in vitro activity is enhanced for dmCBTAU-28.1 (Fig. 5b).

**Fig. 6:**
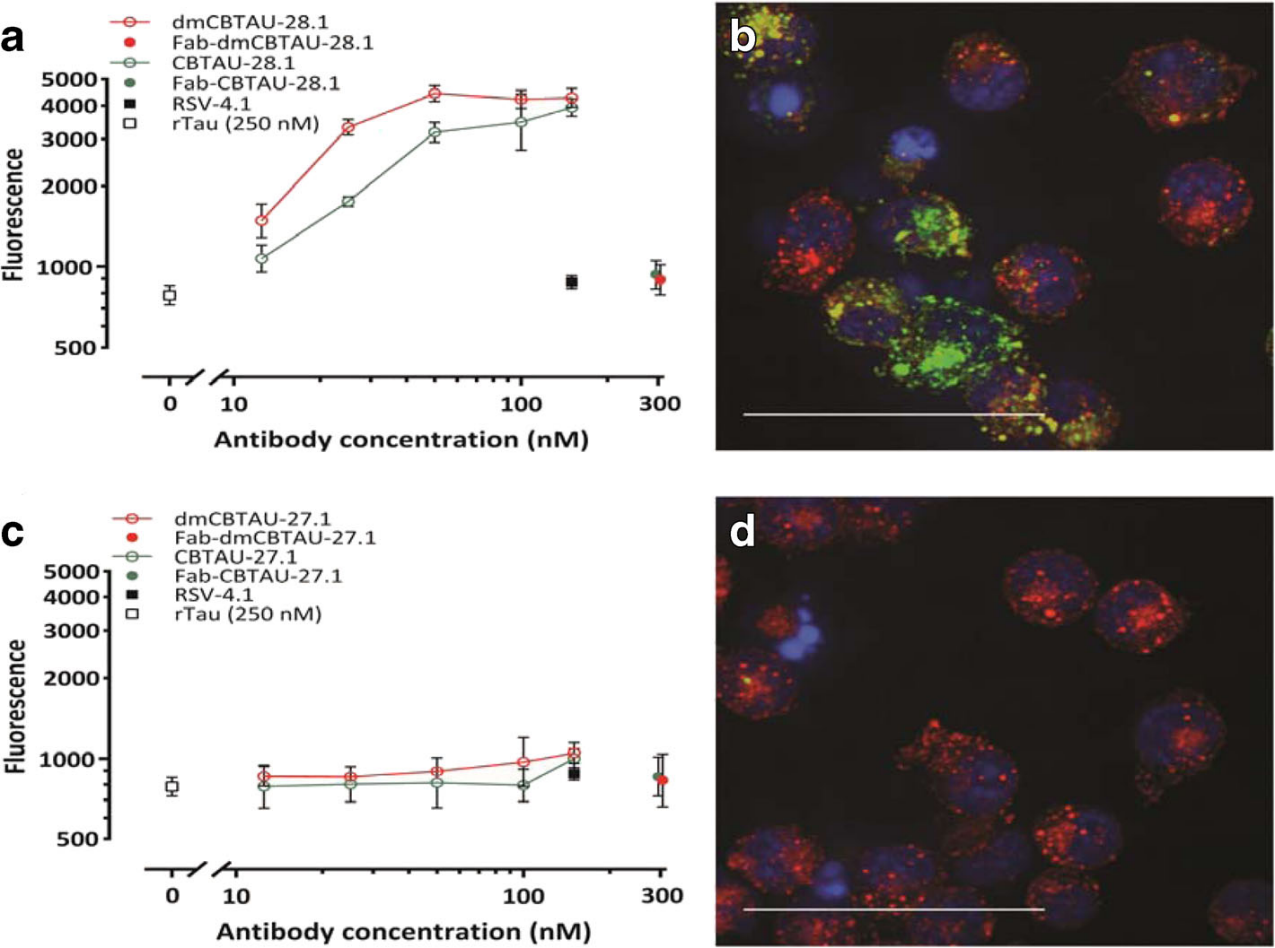
CBTAU-28.1, but not CBTAU-27.1, enhances uptake of tau aggregates into microglial BV2 cells. **a** and **c** Aggregated recombinant tau was covalently labelled with pHrodo Green dye and incubated with chimeric versions (containing mouse instead of human Fc region) of CBTAU-28.1, dmCBTAU-28.1, CBTAU27.1, dmCBTAU-27.1, their Fab fragments, a mouse IgG1 isotype control antibody (IC), or no antibody (rtau). Immunocomplexes were subsequently incubated with BV2 cells and their uptake was assessed by flow cytometry as expressed by the geometric mean fluorescent intensity. Error bars indicate the SD of two independent experiments. **b** and **d** Preformed pHrodo-Green labeled immunocomplexes of rtau with chimeric dmCBTAU-28.1 or dmCBTAU-27.1 (at a concentration of 150 nM) were incubated with BV-2 cells. After incubation, nuclei were stained with Hoechst (blue) and the acidic cellular compartment with LysoTracker Red dye and uptake of immunocomplexes was assessed by live-cell imaging. Images represent maximum intensity projections of a 20 planes Z-stack (0.5 μm planes) acquired with a 63× water immersion objective

The observation that CBTAU-28.1 and dmCBTAU-28 can bind PHFs led us to explore the possibility that these antibodies may furthermore enhance the uptake of tau aggregates by microglia, the resident macrophage cells of central nervous system [17]. Indeed, CBTAU-28.1 and dmCBTAU-28.1 promoted the uptake of aggregated rtau into mouse microglial BV2 cells and the affinity-improved antibody appeared to mediate tau uptake to a greater extent (Fig. 6a). The fact that Fabs of both parental and dmCBTAU-28.1 did not increase basal tau uptake indicates that the uptake is Fc mediated. As expected, CBTAU-27.1 and dmCBTAU-27.1 did not show activity in this assay (Fig. 6c). Antibody-mediated tau uptake and localization of rtau aggregates in the endolysosomal compartment by CBTAU-28.1 and dmCBTAU-28.1, but not CBTAU-27.1 and dmCBTAU-27.1, was confirmed by confocal microscopy (Fig. 6b, and d, and Additional file 1: Figure S14).

## Discussion

### Implications for therapy and vaccines

By binding to the region that is critical for the aggregation of tau and which forms the core of PHFs, CBTAU-27.1 prevents aggregation of tau in vitro. This functional activity identifies its epitope as a potential target for immunotherapy and could perhaps allow earlier intervention than antibodies that inhibit the spreading of already formed tau seeds [6, 42, 48, 49]. Evidence that interfering with tau aggregation through immunotherapy may be possible is provided by murine antibody DC8E8 which also targets monomeric tau, inhibits tau aggregation in vitro*,* and reduces tau pathology in a murine AD model [27]. An alternative approach could be the development of a small molecule drug targeting monomeric tau that interacts with the CBTAU-27.1 epitope. This epitope does not overlap with the microtubule binding motifs (Fig. 1f), suggesting that such a drug may not interfere with the normal function of physiological tau while preventing its aggregation. Furthermore, the epitope of CBTAU-27.1 is specific for tau, and is not present on MAP2, a microtubule stabilizing protein closely related to tau [13]. Like previously described antibodies, CBTAU-28.1 may be able to inhibit the spread of tau pathology. In addition, it demonstrated an Fc-dependent enhancement of the uptake of tau aggregates by microglial BV2 cells. It is probably able to enhance the uptake of aggregates because its epitope is distant from the core of the PHFs and remains accessible.

In summary, CBTAU-27.1 binds an epitope crucial for tau aggregation that becomes buried inside PHFs and therefore inhibits aggregation by binding and sequestering tau. However, it does not decrease seeding activity of previously formed aggregates and is thus functional at earlier stages of tau aggregation. In contrast, CBTAU-28.1 binds PHFs, cross-links tau aggregates and depletes seeding activity, but does not affect initial tau aggregation. CBTAU-27.1 and CBTAU-28.1 (and their respective affinity-improved variants) thus have complementary activities that may allow these antibodies (or drug modalities mimicking these functional activities) to be used for different purposes at different stages of disease. The fact that both antibodies interact with tau aggregates from different tauopathies (Additional file 1: Figure S5) suggests that they may hold therapeutic potential for various related neurodegenerative diseases. Clearly, effective therapy would require binding to, and clearance of different tau aggregate species (e.g. low and high molecular weight species) and assessment of the ability of these antibodies to do so will be the subject of future studies.

Both antibodies are derived from the V_H_5–51 and V_L_4–1 germline families and bind their respective epitopes through hotspot interactions that are remarkably alike, pointing towards a conserved structural motif in tau that could not have been predicted from sequence analysis alone. The motif containing proline and lysine separated by 4 to 7 amino acids, is commonly found in tau, and appears nine times on 2N4R. The lack of somatic mutations around the hotspot proline and lysine suggests these two key tau residues could be responsible for the initial recognition of the V_H_5–51 and V_L_4–1 germline combination. The same V_H_5–51 or V_L_4–1 hotspot interactions can be separately found in other crystallized antibody complexes [7, 19, 33, 36], but the combination of the two germlines could be more prevalent against tau, as they seem to get triggered by the Pro – X_n_ – Lys motif abundantly present on the protein. Identification and characterization of these antibodies may thus be exploited to develop antibody or drug regimens for distinct phases of progression of tau pathology and pave the way towards a peptide-based tau vaccine, by taking advantage of the apparent immunogenicity of the identified motif and presenting both hotspot residues in the right spacing and orientation.

## Additional file

Additional file 1: **Figure S1.** Peptide epitope mapping. **Figure S2.** Reactivity of CBTAU-27.1 and CBTAU-28.1 to PHF-tau. **Figure S3.** Affinity of CBTAU-27.1, CBTAU-28.1 and their affinity-matured mutants for their cognate tau peptides. **Figure S4.** Affinity-improved antibodies dmCBTAU-27.1 and dmCBTAU-28.1 retain both the nature and specificity of the interactions of the parental antibodies with tau. **Figure S5.** Detection of immunoreactivity in various tauopathies by CBTAU-27.1 and CBTAU-28.1 and affinity-improved variants. **Figure S6.** Set-up of an in vitro rtau aggregation assay. **Figure S7.** Complete dataset for in vitro tau aggregation in the absence or presence of CBTAU-27.1 and dmCBTAU-27.1. **Figure S8.** Complete dataset for in vitro tau aggregation in the absence or presence of CBTAU-28.1 and dmCBTAU-28.1. **Figure S9.** Complete dataset for in vitro tau aggregation in the absence or presence of Fab-CBTAU-27.1 and Fab-dmCBTAU-27.1. **Figure S10.** Complete dataset for in vitro tau aggregation in the absence or presence of Fab-CBTAU-28.1 and Fab-dmCBTAU-28.1. **Figure S11.** In vitro tau aggregation in the presence of dmCBTAU-27.1 added at different time points. **Figure S12.** Assessment of CBTAU-27.1 binding to rtau and PHFs by SEC-MALS. **Figure S13.** Macroscopic image of rtau fibrils generated in the absence and presence of CBTAU-28.1. **Figure S14.** Tau aggregates are internalized by BV-2 cells and localize in cellular acidic organelles. **Table S1.** Names and sequences of tau peptides used in this study. The first 10 peptides listed were used as baits in the BSelex method. **Table S2.** Data collection and refinement statistics for CBTAU-27.1 Fab and CBTAU-28.1 Fab. **Table S3.** Data collection and refinement statistics for dmCBTAU-27.1 - A8119 and dmCBTAU-28.1 - A7731 complexes. (DOCX 30952 kb)
