# Supplementary material for: A common antigenic motif recognized by naturally occurring human VH5–51/VL4–1 anti-tau antibodies with distinct functionalities
Source: Acta Neuropathol Commun. 2018 May 31;6:43. doi: 10.1186/s40478-018-0543-z (PMC5984341; doi:10.1186/s40478-018-0543-z)
Supplement: Supplementary file 1 — Figure S1. Peptide epitope mapping. Figure S2. Reactivity of CBTAU-27.1 and CBTAU-28.1 to PHF-tau. Figure S3. Affinity of CBTAU-27.1, CBTAU-28.1 and their affinity-matured mutants for their cognate tau peptides. Figure S4. Affinity-improved antibodies dmCBTAU-27.1 and dmCBTAU-28.1 retain both the nature and specificity of the interactions of the parental antibodies with tau. Figure S5. Detection of immunoreactivity in various tauopathies by CBTAU-27.1 and CBTAU-28.1 and affinity-improved variants. Figure S6. Set-up of an in vitro rtau aggregation assay. Figure S7. Complete dataset for in vitro tau aggregation in the absence or presence of CBTAU-27.1 and dmCBTAU-27.1. Figure S8. Complete dataset for in vitro tau aggregation in the absence or presence of CBTAU-28.1 and dmCBTAU-28.1. Figure S9. Complete dataset for in vitro tau aggregation in the absence or presence of Fab-CBTAU-27.1 and Fab-dmCBTAU-27.1. Figure S10. Complete dataset for in vitro tau aggregation in the absence or presence of Fab-CBTAU-28.1 and Fab-dmCBTAU-28.1. Figure S11. In vitro tau aggregation in the presence of dmCBTAU-27.1 added at different time points. Figure S12. Assessment of CBTAU-27.1 binding to rtau and PHFs by SEC-MALS. Figure S13. Macroscopic image of rtau fibrils generated in the absence and presence of CBTAU-28.1. Figure S14. Tau aggregates are internalized by BV-2 cells and localize in cellular acidic organelles. Table S1. Names and sequences of tau peptides used in this study. The first 10 peptides listed were used as baits in the BSelex method. Table S2. Data collection and refinement statistics for CBTAU-27.1 Fab and CBTAU-28.1 Fab. Table S3. Data collection and refinement statistics for dmCBTAU-27.1 - A8119 and dmCBTAU-28.1 - A7731 complexes. (DOCX 30952 kb) [file 40478_2018_543_MOESM1_ESM.docx]

Electronic Supplementary Material for

**A common antigenic motif recognized by naturally occurring human V_H_5‑51/V_L_4-1 anti-tau antibodies with distinct functionalities**

Adrian Apetri^1*^, Rosa Crespo^1^, Jarek Juraszek^1^, Gabriel Pascual^2^, Roosmarijn Janson^1^, Xueyong Zhu^5^, Heng Zhang^5^, Elissa Keogh^2^, Trevin Holland^2^, Jay Wadia^2,11^, Hanneke Verveen^1^, Berdien Siregar^1^, Michael Mrosek^6^, Renske Taggenbrock^1^, Jeroen van Ameijde^1^, Hanna Inganäs^1^, Margot van Winsen^1^, Martin H. Koldijk^1^, David Zuijdgeest^12^, Marianne Borgers^3^, Koen Dockx^4^, Esther J.M. Stoop^1^, Wenli Yu^5^, Els C. Brinkman-van der Linden^1^, Kimberley Ummenthum^7^, Kristof van Kolen^3^, Marc Mercken^3^, Stefan Steinbacher^6^, Donata de Marco^3^, Jeroen J. Hoozemans^7^, Ian A. Wilson^5,8^, Wouter Koudstaal^1^ & Jaap Goudsmit^1,9,10^

^1^Janssen Prevention Center, Janssen Pharmaceutical Companies of Johnson & Johnson, Archimedesweg 6, 2333 CN, Leiden, the Netherlands.

^2^Janssen Prevention Center, Janssen Pharmaceutical Companies of Johnson & Johnson, 3210 Merryfield Row, San Diego, CA 92121, USA.

^3^Janssen Neuroscience Discovery, Janssen Pharmaceutical Companies of Johnson & Johnson, Turnhoutseweg 30, 2340 Beerse, Belgium

^4^Molecular and Cellular Pharmacology, Discovery Sciences, Janssen Pharmaceutical Companies of Johnson & Johnson, Turnhoutseweg 30, 2340 Beerse, Belgium

^5^Department of Integrative Structural and Computational Biology, The Scripps Research Institute, La Jolla, CA 92037, USA.

^6^Proteros Biostructures GmbH, Bunsenstraße 7a, 82152 Planegg, Germany

^7^Department of Pathology, Amsterdam Neuroscience, VU University Medical Center, De Boelelaan 1117, 1081 HV, Amsterdam, the Netherlands.

^8^Skaggs Institute for Chemical Biology, The Scripps Research Institute, La Jolla, CA 92037, USA.

^9^Department of Epidemiology, Harvard T.H. Chan School of Public Health, 677 Huntington Avenue, Boston, MA 02115, USA

^10^Department of Neurology, Amsterdam Neuroscience, Academic Medical Center, Meidreefberg 9, 1105 AZ Amsterdam, the Netherlands.

^11^Present address: Janssen R&D US, 3210 Merryfield Row, San Diego, CA 92121, USA

^12^ Janssen Vaccines and Prevention, Janssen Pharmaceutical Companies of Johnson & Johnson, Archimedesweg 6, 2333 CN, Leiden, the Netherlands

*Correspondence to: [AApetri@its.jnj.com](mailto:AApetri@its.jnj.com)

**This PDF file includes:**

Figures S1 to S14

Tables S1 to S3

**Figure S1. Peptide epitope mapping.** Binding of CBTAU-27.1 (**A**) and CBTAU-28.1 (**B**) to tau peptides encompassing rtau residues 299-369 and 42-103, respectively, as measured by ELISA.

**
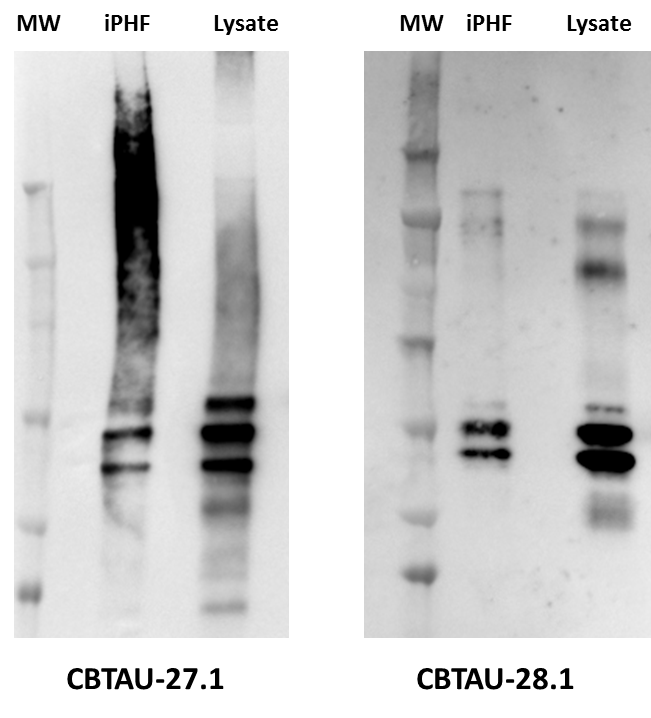
**

**Figure S2. Reactivity of CBTAU-27.1 and CBTAU-28.1 to PHF-tau.** Western blot of CBTAU-27.1 (left) and CBTAU-28.1 (right) with immunopurified PHF (iPHF) and the sarkosyl-insoluble fraction of AD-brain lysates (Lysate). Triple bands characteristic of PHF-tau correspond to approximately 68, 64 and 60 kDa.

**Figure S3. Affinity of CBTAU-27.1, CBTAU-28.1 and their affinity-matured mutants for their cognate tau peptides.** Isothermal Titration Calorimetry (ITC) measurements for CBTAU-27.1 (**A**), CBTAU-28.1 (**B**), dmCBTAU-27.1 (**C**) and dmCBTAU-28.1 (**D**). Variation in enthalpy is observed following incremental addition of mAb stock to tau peptide. Continuous lines represent the best fit of experimental data assuming a single set of binding sites. Experiments were performed in PBS. Equilibrium dissociation constants (*K*_d_) are shown on the individual graphs.

**Figure S4. Affinity-improved antibodies dmCBTAU-27.1 and dmCBTAU-28.1 retain both the nature and specificity of the interactions of the parental antibodies with tau.** Association and dissociation kinetics for the binding of CBTAU-27.1 and dmCBTAU-27.1 to peptide A6897, and of CBTAU-28.1 and dmCBTAU-28.1 to peptide A6940, were determined at different ionic strengths. In accord with the hydrophobic nature of the interaction of CBTAU-27.1 with tau, the ionic strength of the buffer did not significantly affect binding of parental antibody CBTAU-27.1 and its affinity-improved mutant. Given the charged nature of the interaction of CBTAU-28.1 with tau, binding of this antibody was significantly affected by ionic strength of the buffer and also observed for its affinity-improved mutant. For specificity, association and dissociation kinetics for the interactions of the antibodies with a panel of different tau peptides were determined. Lack of significant binding of the affinity-improved mutants to tau peptides to which the parental antibodies did not bind, and to additional peptides not containing the epitopes of the parental antibodies, indicate that the specificity was retained.

**
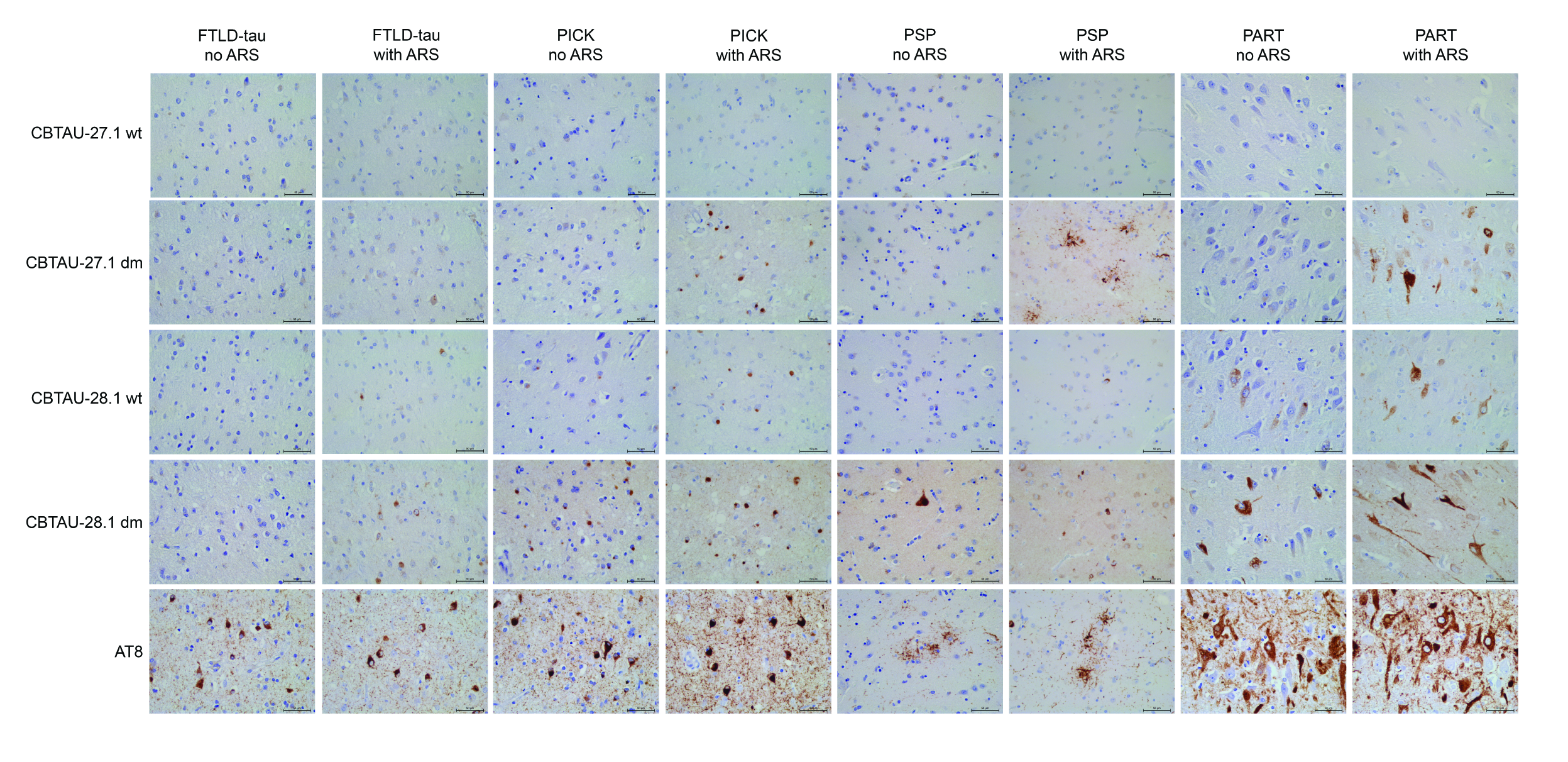
**

**Figure S5. Detection of immunoreactivity in various tauopathies by CBTAU-27.1 and CBTAU-28.1 and affinity-improved variants.** Immunohistochemistry was performed on 5 µm thick formalin-fixed paraffin embedded sections of the hippocampal region using a 0.1 µg/ml antibody concentration without or with heat pretreatment using sodium citrate buffer (antigen recovery step, ARS). Immunoreactivity was visualized using DAB (brown) and nuclei were counterstained with haematoxylin (blue). Representative areas of different tauopathies are shown; frontal cortex for frontotemporal lobar degeneration with tau inclusions (FTLD-tau), frontal cortex for FTLD Pick’s disease (PICK), caudate nucleus for progressive supranuclear palsy (PSP), and hippocampus CA1 for primary age-related tauopathy (PART). Scale bars represent 50 µm.

**Figure S6. Set-up of an *in vitro* rtau aggregation assay.** (**A**) Schematic representation of the nucleation-dependent polymerization process of tau aggregation. The process can be described by an initial energetically unfavorable nucleation phase followed by a fast, energetically downhill growth phase. Addition of pre-formed aggregates (“seeds”) leads to a bypass of the nucleation phase. (**B**) Kinetics for spontaneous rtau aggregation. Aggregation of rtau is induced by addition of heparin in a rTau: heparin ratio of 1:0.5 and continuously monitored by ThT fluorescence (50 μM ThT, excitation at 450 nm and emission at 482 nm) in reaction buffer. The misfolding and aggregation of rtau is a concentration-dependent process. The four independent replicates for each concentration show the high reproducibility of the assay. The obtained aggregates have PHF-like structures as assessed by (**C**) atomic force microscopy (AFM) images of a 15 µM rtau sample taken at 0 (top) and 50 (bottom) hours after initiating of aggregation. (**D**) Kinetics for spontaneous and seeded rtau aggregation in the *in vitro* aggregation assay. For seeding, 2.5 or 0.15 % (v/v) sonicated preformed aggregates were added to fresh rtau monomer solution. Addition of small amounts of pre-formed sonicated fibrillar structures leads to a bypass of the lag phase resulting in long *de novo* generated PHF-like fibrillar structures as observed by AFM. (**E**) Atomic force microscopy (AFM) images of a 2.5% seeded rtau aggregation sample taken at 0 (top) and 10 (bottom) hours after initiation of aggregation.

**Figure S7. Complete dataset for *in vitro*** **tau aggregation in the absence or presence of CBTAU-27.1 and dmCBTAU-27.1.** All four replicates for the spontaneous rtau conversion and each rtau : IgG ratio are shown in separate graphs and the curves that were chosen as representative and shown in Fig. 4a and b are indicated in the same color as they appear in Fig. 4.

**Figure S8. Complete dataset for *in vitro* tau aggregation in the absence or presence of CBTAU-28.1 and dmCBTAU-28.1.** All four replicates for the spontaneous rtau conversion and each rtau : IgG ratio are shown in separate graphs and the curves that were chosen as representative and shown in Fig. 4e and f are indicated here in the same color as they appear in Fig. 4 .

**Figure S9. Complete dataset for *in vitro* tau aggregation in the absence or presence of Fab-CBTAU-27.1 and Fab-dmCBTAU-27.1.** All four replicates for the spontaneous rtau conversion and each rtau : Fab ratio are shown in separate graphs and the curves that were chosen as representative and shown in Fig. 4c and d are indicated here in the same color as they appear in Fig. 4.

**Figure S10. Complete dataset for *in vitro* tau aggregation in the absence or presence of Fab-CBTAU-28.1 and Fab-dmCBTAU-28.1.** All four replicates for the spontaneous rtau conversion and each rtau : Fab ratio are shown in separate graphs and the curves that were chosen as representative and shown in Fig. 4f and h are indicated here in the same color as in Fig. 4.

**Figure S11. *In vitro* tau aggregation in the presence of dmCBTAU-27.1 added at different time points.** *In vitro* tau aggregation (four replicates for each condition) in the presence of dmCBTAU-27.1 at a rtau:IgG ratio of 1:0.6 (purple) added at different time points (hours). For each time point, kinetics was also followed upon addition of equivalent volumes of reaction buffer (black) as control. Time points at which the antibody or reaction buffer were added are indicated by the dotted vertical line in each panel. Data indicate that CBTAU-27.1 interferes with tau aggregation by sequestering monomeric tau, also after initiation of aggregation.

**Figure S12. Assessment of CBTAU-27.1 binding to rtau and PHFs by SEC-MALS.** SEC-MALS analysis of mixtures of antibody and rtau monomers (**A**) or fibrils (**B**), mixed in molar ratio of 1:0.6, incubated at room temperature for 15 min and subsequently centrifuged for 15 min at 20 000 g. The supernatant was taken and injected on SEC-MALS. While binding is observed between the antibody and the tau monomer, as indicated by the shift in retention time of the main peak (**A**), no tau was detected in the supernatant of the tau fibrils sample (**B**), which indicates that it sediment during centrifugation. The majority of antibody was found back in the supernatant of the mixture mAb + fibrils, indicating that the antibody did not bind to the aggregated tau.

**Figure S13. Macroscopic image of rtau fibrils generated in the absence and presence of CBTAU-28.1.** Clumping indicates the capacity of CBTAU-28.1 to inducing large polymeric structures by crosslinking tau aggregates.

**Figure S14. Tau aggregates are internalized by BV-2 cells and localize in cellular acidic organelles.** Preformed pHrodo-Green labeled aggregated rtau/antibody immunocomplexes [chimeric CBTAU-28.1, dmCBTAU-28.1, CBTAU-27.1, dmCBTAU-27.1 and Fab fragments of these; mouse IgG1 isotype control] were incubated with BV-2 cells for 2 hours in the presence of Heparin to block antibody-independent uptake. After incubation, nuclei were stained with Hoechst (blue) and the acidic cellular compartment with LysoTracker Red dye. Live-cell imaging revealed intracellular puncta of pHrodo-Green labeled rtau aggregates inside the cells that were incubated with CBTAU-28.1 and dmCBTAU-28.1, but not with CBTAU-27.1 and dmCBTAU-27.1 or isotype control. Moreover, intracellular rtau often colocalized with LysoTracker Red thus suggesting presence of rtau in the acidic cellular compartment. Images represent maximum intensity projections of a 20 planes Z-stack (0.5 μm planes) acquired with a 63x water immersion objective.

**Table S1.** Names and sequences of tau peptides used in this study. The first 10 peptides listed were used as baits in the BSelex method.

| Name | Residues^1^ | Amino acid sequence^2^ |
| --- | --- | --- |
| A6984^3^ | 1-54 | GSGMAEPRQEFEVMEDHAGTYGLGDRKDQGGYTMHQDQEGDTDAGLKESPLQTPTED |
| A6940 | 42-103 | GLKESPLQTPTEDGSEEPGSETSDAKSTPTAEDVTAPLVDEGAPGKQAAAQPHTEIPEGTTA |
| A6946 | 91-151 | AQPHTEIPEGTTAEEAGIGDTPSLEDEAAGHVTQARMVSKSKDGTGSDDKKAKGADGKTKI |
| A7024 | 139-198 | DKKAKGADGKTKIATPRGAAPPGQKGQANATRIPAKTPPAPKTPPSSGEPPKSGDRSGYS |
| PR5404 | 178-198 | APKTPPSSGEPPKSGDRSGYS |
| A6907 | 186-253 | GEPPKSGDRSGYSSPGSPGTPGSRSRTPSLPTPPTREPKKVAVVRTPPKSPSSAKSRLQTAPVPMPDL |
| A6889 | 241-311 | SRLQTAPVPMPDLKNVKSKIGSTENLKHQPGGGKVQIINKKLDLSNVQSKCGSKDNIKHVPGGGSVQIVYK |
| A6897 | 299-369 | HVPGGGSVQIVYKPVDLSKVTSKCGSLGNIHHKPGGGQVEVKSEKLDFKDRVQSKIGSLDNITHVPGGGNK |
| A6903 | 357-401 | LDNITHVPGGGNKKIETHKLTFRENAKAKTDHGAEIVYKSPVVSG |
| A6914 | 389-441 | GAEIVYKSPVVSGDTSPRHLSNVSSTGSIDMVDSPQLATLADEVSASLAKQGL |
| 2722-2 | 42-61 | GLKESPLQTPTEDGSEEPGS |
| A7731 | 52-71 | TEDGSEEPGSETSDAKSTPT |
| 2722-4 | 62-81 | ETSDAKSTPTAEDVTAPLVD |
| 2722-5 | 72-91 | AEDVTAPLVDEGAPGKQAAA |
| 2722-1 | 82-103 | EGAPGKQAAAQPHTEIPEGTTA |
| A8119 | 299-318 | HVPGGGSVQIVYKPVDLSKV |
| V1158-19 | 309-328 | VYKPVDLSKVTSKCGSLGNI |
| V1158-20 | 319-338 | TSKCGSLGNIHHKPGGGQVE |
| V1158-21 | 329-348 | HHKPGGGQVEVKSEKLDFKD |
| V1158-22 | 339-358 | VKSEKLDFKDRVQSKIGSLD |
| V1158-13 | 349-369 | RVQSKIGSLDNITHVPGGGNK |
| B1014 | 48-77 | LQTPTEDGSEEPGSETSDAKSTPTAEDVTA |
| 2722-5 | 72-91 | AEDVTAPLVDEGAPGKQAAA |
| A7416 | 166-211 | ANATRIPAKTPPAPKTPPSSGEPPKSGDRSGYSSPGSPGTPGSRSR |
| V1085-2 | 36-53 | EGDTDAGLKE**S**PLQTPTE |
| V1091-5 | 192-212 | GDRSGYSSPG**S**PG**T**PGSRSRT |
| B1002 | 299-328 | HVPGGGSVQIVYKPVDLSKVTSKCGSLGNI |
| 386 | 386-409 | TDHGAEIVYKSPVVSGDTSPRHLS |
| P194-212 | 194-212 | RSGYSSPG**S**PG**T**PGSRSRT |

^1^ Number corresponding to residue numbering in 2N4R tau

^2^ Phosphorylated residues are indicated in bold and underlined

^3^ The first 3 amino acids (red) of peptide A6984 are not part of the tau sequence

**Table S2.** Data collection and refinement statistics for CBTAU-27.1 Fab and CBTAU-28.1 Fab

| Dataset | **CBTAU-27.1 *apo*** | **CBTAU-27.1 peptide A8119** | **CBTAU-28.1 peptide A7731** |
| --- | --- | --- | --- |
| **Data Collection** |  |  |  |
| X-ray source | APS 23ID-D | SSRL 12-2 | SSRL 12-2 |
| Space group | C2 | C2 | P2_1_ |
| Unit cell (Å) | *a* = 93.4,  *b* = 60.6,  *c* = 169.5 | *a* = 92.3,  *b* = 59.7,  *c* = 82.3 | *a* = 77.2,  *b* = 49.4,  *c* = 61.4 |
| *β* angle (deg.) | *β* = 103.5 | *β* = 103.6 | *β* = 105.5 |
| Resolution (Å)^a^ | 50.0-1.9  (1.93-1.90) | 50.0-2.0  (2.05-2.00) | 50.0-2.1  (2.14-2.10) |
| Unique reflections^a^ | 72,579 | 27,347 | 25,910 |
| Multiplicity^a^ | 5.7 (3.4) | 4.0 (3.1) | 3.4 (2.4) |
| I/σ(I)^a^ | 17.3 (1.5) | 19.5 (3.5) | 12.7 (1.1) |
| Completeness^a^ | 99.3 (97.0) | 91.5 (61.3) | 97.5 (78.4) |
| *R*_sym_^a,b^ | 0.12 (0.82) | 0.11 (0.38) | 0.12 (0.86) |
| *R*_pim_^a,c^ | 0.05 (0.48) | 0.06 (0.25) | 0.08 (0.65) |
| CC_1/2_ ^a,d^ | 0.998 (0.780) | 0.990 (0.784) | 0.985 (0.714) |
| No. molecules per ASU^e^ | 2 | 1 | 1 |
| *V*_m_ (Å^3^/Da) | 2.3 | 2.3 | 2.3 |
|  |  |  |  |
| **Refinement** |  |  |  |
| Resolution (Å) | 46.53-1.90 | 43.77-2.0 | 41.22-2.10 |
| Reflections (work/free) | 68,813/3,644 | 25,924/1,385 | 24,595/1,314 |
| *R*_cryst_^f^ | 0.186 | 0.168 | 0.244 |
| *R*_free_^g^ | 0.229 | 0.216 | 0.249 |
| Refined atoms  Fab  Peptide  Waters | 6,852  -  637 | 3,398  73  432 | 3,341  85  247 |
| *B*-values (Å^2^)  Fab  Peptide  Waters | 39  -  42 | 24  40  32 | 32  38  30 |
| Wilson *B*-values (Å^2^) | 27 | 20 | 29 |
| Ramachandran statistics (%)^h^ |  |  |  |
| Favored | 97.3 | 97.7 | 96.8 |
| Outliers | 0.5 | 0.0 | 0.4 |
| R.m.s.d. bond length (Å) | 0.007 | 0.005 | 0.007 |
| R.m.s.d. bond angles (°) | 1.12 | 0.94 | 0.95 |
| PDB ID | 6DCV | 6DCW | 5ZV3 |

^a^ Parentheses denote outer-shell statistics.

^b^ *R*_sym_ = Σ*_hkl_*Σ*_i_* |*I_hkl,i_* - <*I_hkl_*>| /Σ*_hkl_*Σ*_i_ I_hkl,i_* and ^c^*R*_pim_ = Σ*_hkl_*[1/(*N*-1)]^1/2^Σ*_i_* |*I_hkl,i_* - <*I_hkl_*>| /Σ*_hkl_*Σ*_i_ I_hkl,i_*, where *I_hkl,i_* is the scaled intensity of the i^th^ measurement of reflection *h*, *k*, *l*, < *I_hkl_*> is the average intensity for that reflection, and *N* is the redundancy.

^c^ *R*_pim_ = Σhkl (1/(n-1))1/2 Σi| Ihkl,i - <Ihkl> | / Σhkl Σi Ihkl,i, where n is the redundancy.

^d^ CC_1/2_ = Pearson Correlation Coefficient between two random half datasets.

^e^ No. molecules for complexes refers to number of Fab molecules per asymmetric unit (ASU).

^f^ *R*_cryst_ = Σ*_hkl_* |*F_o_* - *F_c_*| / Σ*_hkl_* |*F_o_*|, where *F_o_* and *F_c_* are the observed and calculated structure factors, respectively.

^g^ *R*_free_ was calculated as for *R*_cryst_, but with 5% of data excluded before refinement.

^h^ Values are percentage of residues in the favored and outlier regions as analyzed by MolProbity.

**Table S3.** Data collection and refinement statistics for dmCBTAU-27.1 - A8119 and dmCBTAU-28.1 - A7731 complexes.

| Dataset | **dmCBTAU-27.1**  **peptide A8119** | **dmCBTAU-28.1**  **peptide A7731** |
| --- | --- | --- |
| **Data Collection** |  |  |
| X-ray source | PXIII/X06DA^k^ | PXI/X06SA^k^ |
| Space group | C2 | P 2_1_ 2_1_ 2_1_ |
| Unit cell (Å) | *a =* 93.83  *b =* 60.52  *c =* 91.05 | *a =* 50.31  *b =* 63.60  *c =* 141.90 |
| *β* angle (deg.) | *β* =104.4 |  |
| Resolution (Å)^a^ | 88.2-2 .95  (3.20-2.95) | 70.95-2.85  (3.10-2.85) |
| Unique reflections^a^ | 10,377 (2235) | 11,082 (2371) |
| Multiplicity^a^ | 2.8 (2.8) | 3.8 (3.9) |
| I/σ(I)^a^ | 9.5 (2.5) | 10.5 (3.1) |
| Completeness^a^ | 98.0 (98.9) | 99.1 (98.1) |
| *R*_sym_^a,b^ | 0.13 (0.46) | 0.11 (0.45) |
| *R*_pim_^a,c^ | 0.12 (0.43) | 0.10 (0.37) |
| CC_1/2_ ^a,d^ | 0.981 (0.737) | 0.991 (0.875) |
| No. molecules per ASU^e^ | 1 | 1 |
| *V*_m_ (Å^3^/Da) | 2.6 | 2.1 |
|  |  |  |
| **Refinement** |  |  |
| Resolution (Å) | 88.20-2.95 | 70.95-2.85 |
| Reflections (work/free) | 9734 / 643 | 10396 / 686 |
| *R*_cryst_^f^ | 22.0 | 23.8 |
| *R*_free_^g^ | 26.8 | 27.8 |
| Refined atoms  Fab  Peptide  Waters | 3475  89  48 | 3378  92  43 |
| *B*-values (Å^2^)  Fab  Peptide  Waters | 34  46  19 | 44  58  26 |
| Wilson *B*-values (Å^2^) | 32 | 36 |
| Ramachandran statistics (%)^h^ |  |  |
| Favored | 96.0 | 97.5 |
| Outliers | 0.3 | 0.4 |
| R.m.s.d. bond length (Å) | 0.010 | 0.010 |
| R.m.s.d. bond angles (°) | 1.38 | 1.32 |
| PDB ID | 6GK7 | 6GK8 |

^a^ values in parenthesis refer to the highest resolution bin.

^b^ *R*_sym_ = Σ*_hkl_*Σ*_i_* |*I_hkl,i_* - <*I_hkl_*>| /Σ*_hkl_*Σ*_i_ I_hkl,i_* and ^c^*R*_pim_ = Σ*_hkl_*[1/(*N*-1)]^1/2^Σ*_i_* |*I_hkl,i_* - <*I_hkl_*>| /Σ*_hkl_*Σ*_i_ I_hkl,i_*, where *I_hkl,i_* is the scaled intensity of the i^th^ measurement of reflection *h*, *k*, *l*, < *I_hkl_*> is the average intensity for that reflection, and *N* is the redundancy.

^c^ *R*_pim_ = Σhkl (1/(n-1))1/2 Σi| Ihkl,i - <Ihkl> | / Σhkl Σi Ihkl,i, where n is the redundancy.

^d^ CC_1/2_ = Pearson Correlation Coefficient between two random half datasets.

^e^ No. molecules for complexes refers to number of Fab molecules per asymmetric unit (ASU).

^f^ *R*_cryst_ = Σ*_hkl_* |*F_o_* - *F_c_*| / Σ*_hkl_* |*F_o_*|, where *F_o_* and *F_c_* are the observed and calculated structure factors, respectively.

^g^ *R*_free_ was calculated as for *R*_cryst_, but with 6% of data excluded before refinement.

^h^ Values are percentage of residues in the favored and outlier regions as analyzed by MolProbity.

^k^ SWISS LIGHT SOURCE (SLS, Villigen, Switzerland).
